# Behind the screens: perceived impact of COVID-19 on education and the learning environment among school-aged children in the Philippines

**DOI:** 10.1186/s12889-026-27305-4

**Published:** 2026-04-13

**Authors:** Allison Hsu, Jennifer M. Zech, Allison Zerbe, Jyn Allec Samaniego, Cherry Maramag, Mary Christine Castro, Loreto Roquero, Elaine J. Abrams

**Affiliations:** 1 Mailman School of Public Health, ICAP at Columbia University, Columbia University, New York, NY USA; 2 Nutrition Center of the Philippines, Manila, Philippines; 3 Mailman School of Public Health, ICAP at Columbia University, Columbia University, Manila, Philippines; 4 Department of Pediatrics, Vagelos College of Physicians and Surgeons, Columbia University, New York, NY USA

**Keywords:** Philippines, Childhood and adolescence, Academic development, Social development, COVID-19 pandemic, School closures

## Abstract

For school-aged children and their families in the Philippines, nationwide quarantine restrictions implemented due to the COVID-19 pandemic resulted in one of the longest school closures globally; schools re-opened to in-person learning nearly 2.5 years after the initial closure. We aimed to explore the experiences of students aged 8–17 years, their caregivers, and local stakeholders during this time and understand the impact these extended closures had on students’ academic and social development.

From June-November 2023, we conducted focus group discussions (FGD) among students aged 12–17 years and their caregivers and in-depth interviews (IDI) with parent-child (8–11-year-old) dyads. Students were recruited from both public and private schools located in Metro Manila. Key informant interviews (KII) were conducted with local stakeholders, including pediatric health providers, school administrators, and government officials.

A total of 67 students participated in 10 FGDs, 17 caregivers in 4 FGDs, 12 parent-child dyads in IDIs, and 11 stakeholders in KIIs. Students reported facing barriers to remote learning, including at-home distractions and technological challenges. They had trouble adjusting to remote learning formats, which increased their workload while limiting their ability to interact with their teachers and other students. Consequently, they were less motivated to engage with academics and reported feeling that learning effectiveness decreased. Relevant stakeholders observed learning deficits consistent with student self-report. Additionally, students experienced changes in their social-emotional behaviors, becoming more withdrawn from peers but closer with family members. The transition back to in-person learning was difficult, as students felt that school was more challenging than before. Though students felt excited about returning to school and being able to see their friends regularly, they felt uncomfortable participating in class and awkward around their peers. Study participants recommended including curriculum reform as well as support services in schools to assist students’ adjustment back to in-person learning.

Extended school closures led to students feeling less academically adept and ill-prepared to learn new things. Socially, students reported feeling nervous interacting with peers after a long period of isolation. Schools should consider implementing various services to assist in transitioning back to in-person learning to help students feel more comfortable.

**Supplementary Information:**

The online version contains supplementary material available at 10.1186/s12889-026-27305-4.

## Background

The substantial disruption to routines brought on by the COVID-19 pandemic and subsequent lockdown measures has been well-documented [1–5]. The daily lives of primary and secondary school students were especially disrupted as they abruptly pivoted from attending school in-person, engaging face-to-face with their friends and teachers, to logging into virtual classrooms where teachers and classmates appeared only as small windows on their screens. For many students, home was not a conducive environment for learning. Students faced many distractions as parents were often working remotely or tending to the household, siblings were also attending online classes, and students’ attention could be interrupted by easy access to television, online gaming, internet, etc. The sudden transition to remote learning, combined with the broader compounding effects of the pandemic on daily life and wellbeing, posed a myriad of challenges for students that impacted their overall learning experience.

Previous research has shown that challenges faced due to pandemic restrictions and remote learning led to learning loss in school-aged children and adolescents [6, 7]. Learning loss has been defined as “any specific or general loss of knowledge and skills or to reversals in academic progress” [8], and, in the context of COVID-19, has been more recently described as a “decline in K-12 student achievement during the COVID-19 pandemic” [9]. Learning loss is observed in students during regular vacation breaks. It has been reported that the loss that occurs over summer vacation is equivalent to one month of learning [10]. Learning gaps tend to be larger in younger than older students for the same amount of time not in school [11]. This emphasizes the fragility of younger students and the importance of maintaining stability with their education in order to ensure they are getting the most out of learning. There is also evidence that pandemic restrictions, especially social distancing that necessitated increased virtual interaction with peers, have had mixed effects on the development of children’s social skills. Some adolescents have reported more mental health issues and others describe closer relationships with their peers despite physical isolation [5, 12].

The Philippines, in particular, had a unique experience with school closures due to pandemic restrictions. Schools were closed to in-person learning for over two and a half years, representing one of the longest school closures globally [13]. Further, even prior to the pandemic, the country had been managing significant and distinct challenges, including worsening seasonal typhoons [14] that devastated limited infrastructure, weakened already poor internet service, and brought on substantial financial challenges [15]. The Philippines had also documented existing low learning levels prior to pandemic restrictions. According to the 2018 Programme for International Student Assessment, which assesses 15-year-olds in mathematics, science, and reading, the Philippines ranked 78th of 78 countries in 2018. In 2022, the country still ranked low, 77th of 81 countries, with scores 100 points below the Organisation for Economic Co-operation and Development average in both years [16].

At the time of our study, literature coming out of the Philippines on the impact of pandemic restrictions was limited and largely commented on the physical and mental health of the Filipino general population. Few studies evaluated the impact of the restrictions on students’ academic development, and, of these, most focused on a single cadre of respondents rather than multiple stakeholder groups [2, 17, 18]. The prolonged school closures and resulting extended pause in students’ in-person learning make the Philippines a unique and important setting in which to examine the impacts of the remote learning environment on the academic performance and social behaviors of children and adolescents. To achieve this aim, we conducted a qualitative study among students aged 8–17, their parents/caregivers, and local stakeholders to gain greater insight into personal experiences during remote learning and the transition back to in-person schooling as well as assess the impacts these experiences had on students’ academic and social development. We also report on the recommendations that participants gave to address the continued impact of COVID-19 and the school closures on learning.

## Methods

### Study design & setting

Between June 2023 and November 2023, we conducted a qualitative study with students, parents/caregivers, and local stakeholders in Metro Manila, Philippines. As the largest metropolitan area in the Philippines, Metro Manila included a large number of schools from which to draw our sample and also offered access to a diverse set of schools that allowed for collection of a wide range of student and parent/caregiver experiences. Students aged 12–17 years and their parents/caregivers were recruited for focus group discussions (FGD), while students aged 8–11 years and their parents/caregivers were recruited as child/parent dyads for in-depth interviews (IDI). The broad age range was intentionally selected to capture differences in students’ experiences across different age groups and developmental stages. FGDs were used with older students and caregivers as a way to facilitate interactive discussion and collective reflection, allowing participants to build on shared experiences in a relaxed environment [19]. IDIs with child/parent dyads were conducted with younger students and their caregivers to support children’s ability to participate meaningfully given their age, communication skills, and need for parental support. Local stakeholders recruited for key informant interviews (KII) included educators from both public and private schools, healthcare providers (psychologists, psychiatrists, pediatricians), local researchers, child health and mental health advocates, and policy makers. These stakeholders were included in order to collect data on experiences with implementing pandemic-related policies as well as any plans to address the emerging needs of the pediatric population in response to the effects of the pandemic.

This study was conducted in partnership with a local non-profit organization, Nutrition Center of the Philippines (NCP), who has extensive experience conducting qualitative research studies with school-aged children, as well as connections with both government and non-government sectors.

### Description of COVID closures in the Philippines and school settings

Schools closed in the Philippines in March 2020 shortly after the World Health Organization declared COVID-19 to be a worldwide pandemic [17], disrupting education for nearly 25 million students [20]. After a period of no school between March and October 2020 [21], remote learning was introduced through online classes using digital platforms and/or modular lessons with printed materials distributed and collected by teachers [20, 22].

**Fig. 1:**
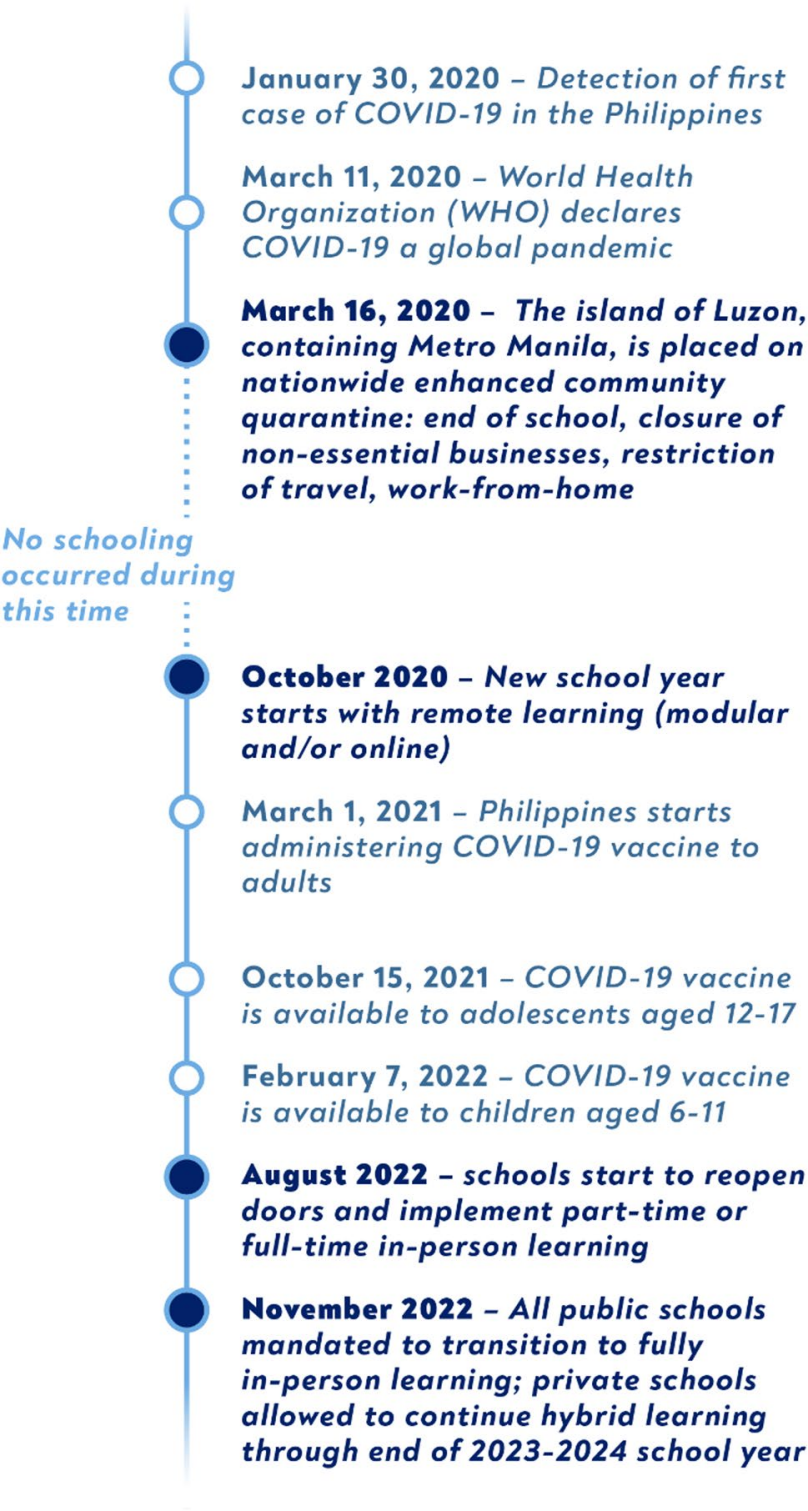
Timeline of COVID-19 pandemic school closures and re-openings

In August 2022, after 29 months, schools started to re-open to in-person learning, though many public schools continued hybrid learning until November 2022 and private schools were permitted to do so through the 2022–2023 and 2023–2024 school years [22, 23]. The Philippines was one of the last countries in the world to go back to in-person learning, experiencing an extended period of educational disruption [13, 21, 22]. Figure 1 gives a visual presentation of the relevant events that occurred. Our data collection occurred about one year after schools fully re-opened to in-person learning.

### Sampling & recruitment

Sample size was informed by prior experience with achieving thematic saturation in similar qualitative studies and was designed to be feasible within the study timeline and budget. We aimed to recruit 96 students aged 12–17 for 12 FGDs with 8 students each, 24 parents/caregivers for 3 FGDs with 8 parents each, and 12 child/parent dyads. We also aimed to recruit 12 stakeholders for the KIIs. Purposive sampling was used to select respondents for this study. For parent and student data collection activities, a list of schools was prepared to identify potential study sites. This list was created based on the type of school: (a) public, or (b) private; and the mode of learning the school offered during school closures: (a) full modular, (b) online/virtual learning, or (c) a combination of both modular and virtual learning. The schools identified also needed to be hosting students in-person at the time of the study, whether that was full-time or hybrid. Both public and private schools were included to account for differences in school-level quarantine guidelines and support given to students. NCP then met with the Department of Education (DepEd) to introduce the study and proposed list of sites and request a formal letter of endorsement to allow public schools to participate. With this endorsement letter, NCP then liaised with leadership at the pre-identified schools to coordinate study activities. For private schools, NCP sent a letter of request directly to school heads.

Following this approval process, students were then recruited with a letter of intent and a copy of the informed consent form to share with their parents. Those who expressed interest were asked to notify their teachers or contact the study team directly to complete the informed consent process and schedule a time for conduct of FGD/IDI. Students were eligible for participation if they were (1) currently attending school in-person and (2) received parent/caregiver consent. Students provided written informed assent and caregivers provided written informed consent before any student participation in study activities. Parents and caregivers were eligible for their own focus group discussions if they were (1) a caregiver for a child or children currently enrolled in school and who was also enrolled during the 2020–2021 and 2021–2022 school years, and (2) had provided consent for their child to take part in the study.

For KII, a list of potential stakeholders was prepared using publicly available information from agency websites and assistance from personal contacts. Leadership from schools selected for FGDs/IDIs were also targeted for the KIIs. Eligibility criteria for stakeholders included having previous or current work or academic experience in children’s health or education or policies relating to the COVID-19 pandemic lockdown.

### Data collection

All study activities were conducted by experienced and trained local researchers and study materials were translated into Filipino and back translated to English for accuracy. Guides for all study activities were piloted among the research team.

The guides for the FGDs and IDIs were developed based on a literature review of other studies exploring the impacts of COVID-19 on students. Domains discussed during FGDs and IDIs included the impact of COVID-19 pandemic-related school closures on physical health, mood, behavior, relationships with friends and family, daily activities, and risk-taking behavior. Participants were asked to share their experiences with remote learning and the transition back to in-person learning. The questions across age groups did not differ significantly. Questions on the IDI guide for younger children (aged 8–11 years) were revised from the FGD guide only to be more age-appropriate, using narrative storytelling and visual tools to engage with the child. These techniques were researched in advance to ensure that vignettes developed to describe situations were both age and culturally appropriate for Filipino children. The parents/caregivers were asked about their child’s experiences as well as their own. FGDs lasted between 1 and 3 h, while IDIs were approximately 1 h.

KIIs explored the impact of COVID-19 pandemic-related policies and school closures on students, caregivers, and teachers, as well as thoughts around the transition back to in-person learning. KII participants were also asked about their thoughts about the needs of the school-aged population, their caregivers, and teachers in the Philippines. KIIs with stakeholders lasted about 1 h.

### Data analysis

All study activities were audio-recorded with the assent/consent of the participants. Recordings were transcribed and translated into English from Filipino by study team members fluent in both languages. Transcripts were analyzed using an inductive thematic approach, with line-by-line coding conducted iteratively until saturation was reached, following analytic guidance by Creswell & Poth [24]. Codes were generated directly from the data and refined through repeated review and comparison across transcripts. A structured coding scheme was developed and applied consistently to all transcripts. One primary researcher conducted the preliminary coding, then convened a team to discuss codes and emerging themes as well as resolve ambiguities, in order to strengthen interpretation and credibility. Data was analyzed using QSR Nvivo (v.1.7.1, Doncaster, Australia). The team identified 85 codes across multiple rounds of coding.

### Ethical considerations

The study protocol was approved by the Columbia University Medical Center Institutional Review Board (AAAU5835) and the St. Cabrini Medical Center-Asian Eye Institute Ethics Review Committee (ERC# 2023-009). Administrative permission to enter schools was granted by the Department of Education and the principals of each institution. Student participants provided written informed assent and caregivers provided written informed consent prior to participation in FGDs and IDIs. Written consent for KIIs was waived as stakeholders may not be able to meet in person. They instead were given the option to provide consent electronically. All informed consent and assent forms were provided in both English and Filipino. All study activities were conducted in accordance with the Declaration of Helsinki.

## Results

### Participant and study setting characteristics

We conducted 5 FGDs among 12-14-year-olds and 5 FGDs among 15-17-year-olds, 4 FGDs among parents/caregivers of students, and 12 parent/child dyad IDIs (Table 1). We also conducted 11 KIIs with stakeholders including policymakers/members of local government units (2), national government agency representatives (2), school representatives (5), non-school-based psychologists (2).

**Table 1:** FGD and IDI participants

	# of schools	8-11-year-olds IDIs (# of participants)	12-14-year-olds FGDs (# of participants)	15-17-year-olds FGDs (# of participants)	Parents - FGDs (# of participants)	Parents – IDIs (# of participants)
Public school	3	6 (6)	3 (24)	3 (22)	2 (11)	6 (6)
Private school	2	6 (6)	2 (8)	2 (13)	2 (6)	6 (6)
Total	5	12 (12)	5 (32)	5 (35)	4 (17)	12 (12)

The students who participated in the study were similar across public and private schools: both groups had a median age of 14 years and male and female students participated almost equally. The majority of the parents/caregivers who participated in the FGDs were female (27/29, 93%). Caregivers of students in private school were more likely to be employed and had completed more years of formal education compared to parents of students in public school (Table 2).

**Table 2:** Demographics of participants in IDIs and FGDs

Students	Public school (*N* = 52) (%)	Private school (*N* = 27) (%)	Total (*N* = 79) (%)
8–11 years old (N)	6	6	12
12–14 years old (N)	24	8	32
15–17 years old (N)	22	13	35
Age – median (interquartile range)	14 (13,16)	14 (13,16)	14 (13,16)
Gender			
Male	23 (44%)	13 (48%)	36 (46%)
Female	29 (56%)	14 (52%)	43 (54%)

**Parents/caregivers**	Public school (*N* = 17) (%)	Private school (*N* = 12) (%)	Total (*N* = 29) (%)
Age – median (interquartile range)	44 (23, 59)	44 (35, 49)	45 (39, 49)
Gender			
Male	1 (6%)	1 (8%)	2 (7%)
Female	16 (94%)	11 (92%)	27 (93%)
Employment status			
Employed	8 (47%)	11 (92%)	19 (66%)
Official of government and special interest organization	3 (38%)	1 (9%)	4 (21%)
Professional	3 (38%)	9 (82%)	12 (63%)
Office worker	1 (13%)	1 (9%)	2 (11%)
Working class	1 (13%)	0 (0%)	1 (5%)
Unemployed	9 (53%)	1 (8%)	10 (34%)
Education level			
Junior high school (grades 7–10)	6 (35%)	1 (8%)	7 (24%)
Senior high school (grades 11–12)	0 (0%)	0 (0%)	0 (0%)
Vocational secondary school	1 (6%)	1 (8%)	2 (7%)
Undergraduate	7 (41%)	5 (42%)	12 (41%)
Postgraduate	3 (18%)	5 (42%)	8 (28%)

The majority (80%) of participating schools serve low- to middle-income families in Metro Manila. Private schools notably serve middle- to high-income families, with one school requiring high tuition fees. All schools were closed for 29–30 months as required by quarantine restrictions (Table 3).

**Table 3:** Characteristics of schools participating in the study

School	Type of school	Number of months closed	Student characteristics
1	Public	29 mos	Low- to middle-income families
2	Public	29 mos	Low- to middle-income families
3	Public	29 mos	Low- to high-income families Specialized public school
4	Private	29 mos	Low-, middle-, and high-income families Offers low-cost or free tuition
5	Private	30 mos	Middle- and high-income families High tuition fees

### Main findings by theme

**Fig. 2:**
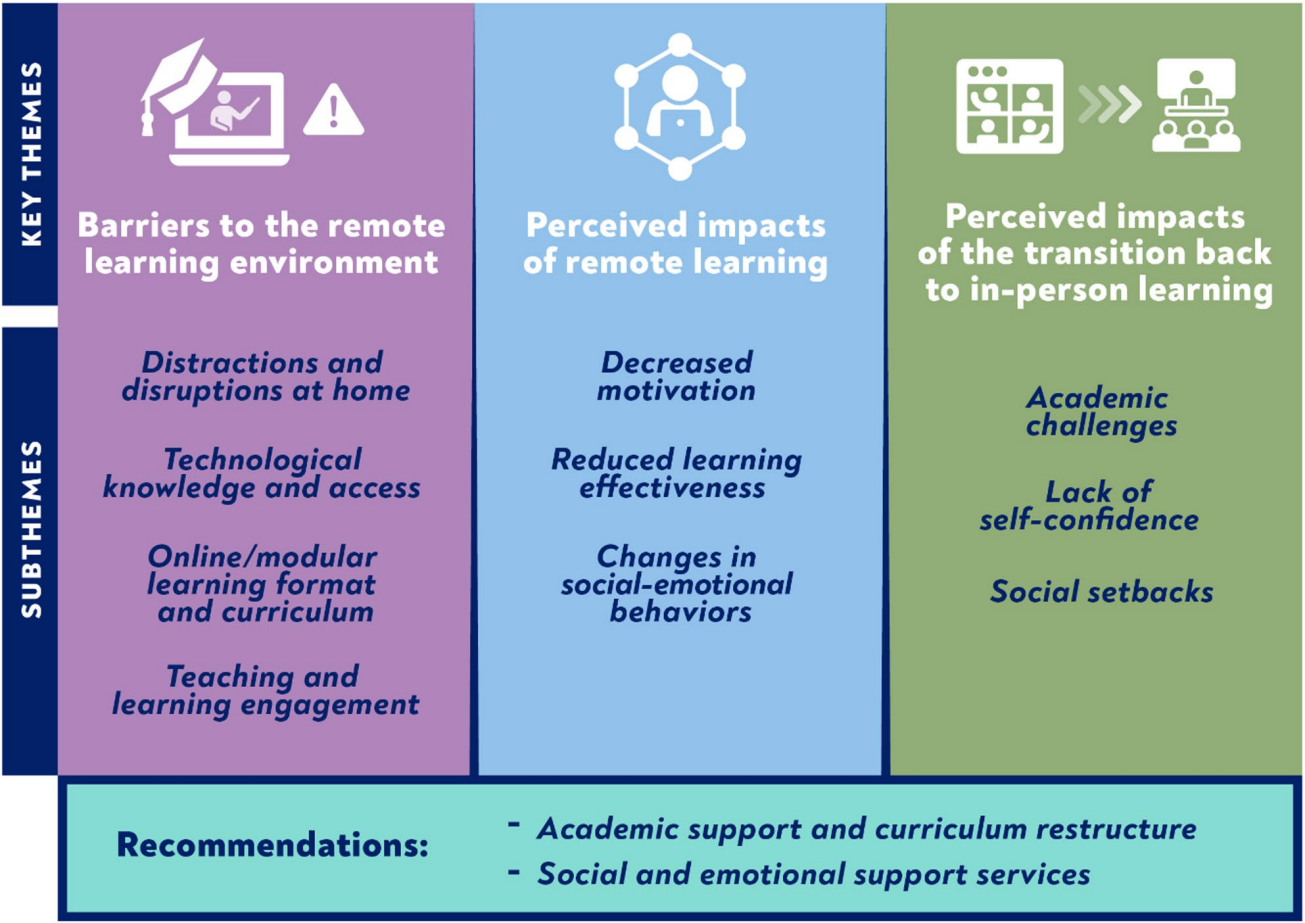
Key themes: Barriers and impacts of changed learning environment among students, caregivers and stakeholders

Below are the main findings, with supporting quotes, describing the perceived and experienced barriers and impacts of remote learning due to COVID-19 for students as well as their caregivers and teachers, categorized by key themes. We did not discern any gender differences in participant perspectives across the different groups. Likewise, students in both public and private schools shared similar experiences. Figure 2 shows an overview of emergent key themes.

### Barriers to the remote learning environment

The transition from in-person to remote learning at the start of lockdown was met with many challenges such as disruptions at home, challenges with technology and online learning format, and reduced engagement, creating barriers to effective learning.

#### Distractions and disruptions at home

When schools abruptly closed to comply with quarantine restrictions, students went over half a year with no schooling and then were required to adapt quickly to fully remote learning at home when the new school year started. Homes became crowded, shared spaces where multiple family members worked or studied simultaneously. Students reported experiencing many distractions and disruptions within their homes that impacted their learning, such as increased interactions with family members, household chores, and expanded access to the internet and television. Older students (aged 12 + years) were often asked to help around the house, taking time and attention away from their schoolwork. Because students were home-bound, the balance between schoolwork and housework was difficult to maintain.

“School works pile up because we also had a lot of household chores. I can no longer prioritize my schoolwork because I’m busy at home.” − 12–14-year-old student, male, public school.

“You do the homework. Then you will also do the household chores. You have lack of sleep.” − 15–17-year-old student, male, public school.

Students also had increased access to their computer and internet during school hours, making entertainment such as YouTube, games, and television more readily available, further reducing time and attention allocated to schoolwork.

“I am watching or scrolling on TikTok when I become distracted. It is fun… until someone calls you for recitation.” – 12-14-year-old student, female, public school.

“That is one negative effect of the pandemic. I used to limit their time with gadgets but since the pandemic, I needed to extend them. That’s the negative effect. They were exposed to gadgets.” – mother, public school.

#### Technological knowledge and access

Local government units (LGU) that deliver public services to communities at the grassroots level, as well as public schools were responsible for providing resources to support remote learning, such as printed materials for modular learning. Several LGUs were even able to provide tablets to public school students. However, not all families had access to the necessary devices or internet at home, often requiring out-of-pocket expenses to participate in remote learning. In households with multiple children participating in remote learning, electronics often had to be shared creating challenges when siblings had simultaneous synchronous classes.

“We are three siblings, so sometimes, if one of my siblings and I had concurrent classes, that’s where we had difficulty using the tablet.” – 15-17-year-old student, male, private school.

When electronics were available, accessing and maintaining a high-quality internet connection was a major barrier for students. Many students often complained about laggy videos and poor connection during synchronous classes, hindering their abilities to understand the lessons being taught.

Several students reported that they and their teachers struggled with the transition to remote learning due to varying levels of technological knowledge and inconsistent use of online platforms. Most teachers were unfamiliar with the online platforms required for remote learning, and adapting to this new technology was burdensome. Not all teachers or classes used the same system, requiring students to navigate multiple platforms to access lessons, submit assignments, and communicate with their teachers. This inconsistency led to confusion and placed additional pressure on students, who had to juggle learning new technologies on top of their regular schoolwork and often provide technical support to their teachers.

“But a little bit confusing *[on the use of online platforms]*, because it depends on the teacher what kind of communication they use, there is Discord, or Zoom, sometimes the submission is on [*Facebook*] messenger.” – 15-17-year-old student, male, public school.

“Some of our teachers were old so, they’re not techy, sometimes they get confused in the meetings, sometimes they’re gonna ask us, “How to do this one?“, “Which one should I click?“. I have a teacher who doesn’t really know how to use Zoom, so I really struggled with his subject because no one was teaching.” – 15-17-year-old student, female, public school.

#### Online/modular learning format and curriculum 

Regular school schedules and curriculums were modified to accommodate learning in the context of the pandemic. Schools reduced the length of class time to give screen breaks, and curriculums were modified to only include essential subjects. This meant supplementary schoolwork needed to be completed by students on their own time versus during school hours.

“Before the pandemic… giving of assignments on Friday was not allowed. But when it shifted to online, it no longer applied… everything was compressed.” – mother, private school.

Many students noted an increase in school activities and assignments as compared to in-person learning. This was attributed to teachers’ efforts to keep students accountable and engaged without the ability to observe students in person or consistently via video, as many schools did not require camera use during class.

“Personally, we can’t force them and we can’t see them physically and on camera while they can see us [*teachers*] since we have our cameras turned on. Most of the efforts came from us teachers though they also gave theirs.” – grade level guidance teacher, public school, female.

Parents had to become more involved in helping their children with their schoolwork during remote learning as student workloads increased and became more difficult. Stakeholders commented that many parents and caregivers were limited in the amount of support they were able to provide their children due to lack of knowledge of teaching methods and the material itself.

“It’s really hard because I don’t even know what the answer is. The questions in the modules are really hard. I even asked myself, ‘how to do this?’, ‘I didn’t study this before’, that’s what always came to my mind.” – mother, public school.

“From the student’s perspective, they are sad since their space is limited, and the parent’s patience is not the same as their teachers… The child-to-parent instruction is different from the teacher since they are knowledgeable about the psychology and learning style of the students.” – public school principal, female.

#### Teaching and learning engagement

Engagement challenges arose with remote learning which presented a new environment for teacher-student interactions. Students reported feeling disconnected from their teachers as the lack of face-to-face interaction limited opportunities to ask questions in real time. From many students’ perspectives, teachers were not explaining concepts fully, making it difficult to understand lessons or complete their homework. Some students also reported that they felt teachers were inaccessible outside of schooltime and were often not comfortable asking questions or reaching out to teachers for support. Instead, many students tried to teach themselves using the internet as a resource.

“Stressed…Because sometimes it’s hard and the teacher doesn’t explain it.” – 8-11-year-old student, female, private school.

“It was also difficult because I couldn’t ask the teacher directly because I was shy, if I had a question, I would just search it on the net… we couldn’t approach them, so we just searched for the answers.” – 12-14-year-old student, male, private school.

The growing disengagement between students and teachers was observed by parents and stakeholders as well.

“Because the prep [*preschool student*] doesn’t know how to read yet, then they have a Zoom meeting, they just do the lesson on their own, the teacher will just give instructions, so it’s difficult.” – mother, private school.

“So, for example, they have difficulty with the lesson they just won’t submit or they won’t even comply. They are shy to consult with the teachers… thinking that we are all having a hard time with this online learning so… why would I bother my teachers with this my own experience or with my own difficulty?” – guidance counseling unit head, public school, female.

### Perceived impacts of remote learning

The combined effects of the barriers to remote learning as described above profoundly impacted both students’ learning abilities and progress. Students reported their decreased motivation to learn and felt they were not learning as effectively as they had in a traditional classroom setting, a concern echoed by caregivers and other stakeholders. In addition to academic struggles, remote learning also influenced students’ social behaviors, which are explored further below.

#### Decreased motivation

Distractions around the house, the lack of in-person accountability, as well as the new, monotonous forms of learning decreased students’ focus and motivation to participate in class.

“Sometimes you just actually… you’re not learning anymore. You’re just striving. You just want to pass only all the requirements and that’s the consequences of this pandemic.” – 15-17-year-old student, female, public school.

“My child is more active during face-to-face classes and does her assignment immediately after arriving home. Compared to remote schooling, my child did not have a desire to study.” – mother, public school.

Some students described often feeling a sense of laziness and disinterest during remote learning, recounting that they lacked the same energy or excitement they once had for school.

“I mean it’s not hard but…I don’t have motivation. I don’t want to do them [*assignments*]. But I know I can do them. But I just don’t do it…[*was*] lazy.” – 8-11-year-old student, female, private school.

Despite challenges to remote learning and general decreased motivation, several students reported that they were determined to do well on their own.

“My academic performance increased since I was determined to finish this on my own.” – 12-14-year-old student, male, public school.

#### Reduced learning effectiveness

Many students reported that they struggled to retain what they were learning, leading to an overall decline in perceived learning effectiveness during remote schooling. Members of the youngest age group mentioned that not seeing their classmates during remote learning negatively affected their educational experience, as opportunities to help each other as they did during face-to-face schooling were basically nonexistent in the online setting.

“When I’m at home I can’t study because I don’t understand what I’m reading, but at school I understand. Because I also have seatmates at school that I go with.” – 8-11-year-old child, male, public school.

Older students felt that remote schooling limited them to surface-level learning, preventing the deeper understanding they typically experienced during in-person classes.

“It got worse because I used to be in honors, and now I’m like an average student… The reason why I got worse was because I had a hard time understanding the online class because it seems like you’re just staring at the screen. It’s really hard if you’re studying. You don’t feel like they are teaching you. If they are teaching, you were just watching.” – 15-17-year-old, male, private school.

Several older students expressed concerns that their decrease in learning during remote learning could have long-term consequences for their academic progress.

In contrast, only a small number of participants mentioned that they felt they learned more during remote learning, especially with independent study.

“In self-learning, your knowledge is increased. It’s not like you always depend on a teacher. As we keep on relying on the teacher, sometimes we only set our boundaries on what is taught.” – 15-17-year-old student, female, public school.

Parents observed a clear decline in their children’s learning during remote learning, and many were able to identify specific skills their children were struggling to develop or had fallen behind in.

“There’s one, he is getting lazy writing. They really don’t like to write unlike before.” – mother, public school.

Similarly, stakeholders in the education sector noticed that young students were behind in reading and writing skills and older students were lacking the basic knowledge needed to build upon to advance to higher grade levels.

“Their reading assessment, they have many deficiencies. Their penmanship is not good enough… Since reading is a tool to improve the learning outcome of the students, if there is a problem in reading, the comprehension is missing, and it will affect other subject areas.” – public school principal, female.

“For example, their competency of Grade 8, you were absent for a long time, which can tell that you may not be able to master the competency for Grade 7, which is a bad thing. If the level of the student is growing, there are a lot of competencies to meet. So, as the students grow older, the challenges and quality is increasing too. Therefore, that was so called the learning gaps or now it was called the learning loss.” – DepEd NCR representative, female.

#### Changes in social-emotional behaviors

Students reported changes in their social behaviors and relationships due to being at home all the time. Parents noticed that their children started to struggle with mental health issues due to the restrictions and circumstances of the pandemic overall. This shift not only affected students, but also caregivers and teachers who experienced stress during this time.

Students spent much more time at home during remote learning and were able to build stronger relationships with family members due to the quarantine restrictions.

“Having more time with my family at home. Ah. Happy.” – 8-11-year-old student, male, private school.

“Me and my family became closer. Before, we did not have a bonding but when the pandemic came, we only stayed under one roof and could not go out so we had open communication that we developed there.” – 12-14-year-old student, male, public school.

The shift to online learning left students feeling distant from their classmates and peers. They struggled with the absence of in-person interactions, making it difficult to maintain connections and a sense of community.

“To be honest I had a hard time adjusting because I’m used to face-to-face classes with my friends but when we shifted to online via phone/internet, it was like they are different people/strangers.” – 12-14-year-old student, female, public school.

However, some students reported taking advantages of online games and social networks to grow closer to peers.

“But the good thing there is [*Facebook*] messenger and Roblox, using that application I can talk with them.” – 8-11-year-old student, female, private school.

Parents noted that the stress of the conditions of the pandemic may have caused some mental health issues in their children.

“In the first year he was fine but then in the second year there are a lot of red flags such as he never eats with us… he is always in his room and sleeping. Then I realize that it is the one that we’ve talked about in the mental health seminar. He is always sleeping? Check. Not eating much? Check. Not taking a bath? Check. We get to know it through the webinar and it’s really a great help.” – mother, public school.

“There was a psychologist, a parent from a higher year level that meets her online every week for 10 weeks. Findings showed that her grieving was caused by the death of her classmate and also due to the pandemic.” – mother, public school.

The at-home learning environment also caused tension in parents, who now had to watch their children full-time and take part in their learning, while also managing their own adjustments to the pandemic and the enduring restrictions.

“Actually, I am struggling with the changes happening because it is not like we used to. Your time is really changing because your attention will be divided, we have work and then your attention to work is divided to your child. You can’t focus really because of work.” – mother, public school.

“And I think our caregivers are possibly not that aware of how to handle the concerns of their children or those they care for during the pandemic…How can I talk to my son so that he understands what the pandemic is? When my son tells me that I’m sad, or he is sad, how should I process him? How to deal with the mental health concerns of their children, of those they take care of?” – psychologist, NGO employee, female.

Teachers also faced the stress of having to transition to remote learning, while at the same time dealing with and processing the pandemic and its implications in their own lives.

“Personally, the sense of routine is mixed up because I should just be at home, I’m resting. All of a sudden you have to work from home.” – psychologist, NGO employee, female.

“I see it as harder because there are no restrictions. You should always be online…” – grade level guidance teacher, public school, female.

### Perceived impacts of the transition back to in-person learning

Students were generally excited to go back to in-person learning, especially to see and learn with their friends, though some felt nervous. The return to a structured schedule required adjustment, and many students continued to face academic challenges, feeling behind in their studies. Social behavior changes were observed, including reduced self-confidence and setbacks in peer interactions and socialization.

#### Academic challenges

Students in both the 12-14- and 15-17-year-old groups expressed that schoolwork was more difficult after returning to in-person learning than before the pandemic. They felt that they lacked knowledge they should have acquired and noted that assignments had increased in both difficulty and quantity.

“Assignments nowadays are more difficult. Since our grade level is increasing. Back then, teachers were not providing a lot of assignments but now there are plenty of assignments given.” – 12-14-year-old student, female, public school.

“Chemistry was also difficult today, because we all missed chem 1 and chem 2, so when we come for chem 4, sometimes the teachers will say that we did it in grade 9, but we didn’t remember because it was online.” – 15-17-year-old student, male, public school.

A number of students also felt behind in their learning, feeling that they were constantly playing catch-up and could not meet academic expectations.

“Rush… there are many things to catch up. No time for your new lesson because it is pure catch up.” – 12-14-year-old student, male, public school.

“…it became more challenging because… it’s been two years in online class and just few knowledge we acquired from it. So, it seems like that our knowledge is cut off… [it has] become harder to meet the expectation… because they thought that we had a higher grade in online class.” – 15-17-year-old student, female, public school.

#### Lack of self-confidence

After the transition back to in-person learning, students felt uncomfortable participating in class and had growing concerns about the difficulty of their schoolwork and the consequential effect on their grades.

Students in the 12-14-year age range shared that they did not feel confident talking in front of the class, as they had grown out of practice during remote learning.

“The interaction with the large crowd is a problem and the confidence in reporting was lost. I felt nervous since during online class there are few reports. And some people have a fear of the crowd and it is very troublesome to do reporting because you need to modulate your voice. But we still do reporting for the grades.” – 12-14-year-old student, female, public school.

One mother recounted some teachers’ feedback that students were reluctant to participate in class.

“She became shy. Even her older brothers were not active in school when they went back to face-to-face classes because they needed to be accustomed to interacting with their classmates. That’s also what the teachers told us…they noticed that the children were inactive. Unlike before, children always participate in recitation. Now, if they would not call the students during recitation, they wouldn’t answer.” – mother, public school.

Students also described their anxiety about their grades as well as the amount of work that came with returning to in-person learning, adding to their uncertainty of being able to perform well academically.

“[*feeling*] Lack of confidence and being scared of the grades.” – 12-14-year-old student, female, public school.

#### Social setbacks

Despite the excitement that students felt about going back, once in school, students reported feeling awkward, nervous, and shy when it came to socializing with their peers.

“I no longer know my classmates.” – 8-11-year-old student, female, public school.

“It was still okay but after COVID, it became awkward for us to interact with one another, others became shy to approach others too.” – 15-17-year-old student, female, public school.

Students were worried about judgement of their changing appearances during their teenage years, especially since classmates had not seen each other consistently in over two years. Some students expressed that their self-esteem had decreased over the course of the quarantine restrictions.

“Adjusting to our physical appearance, I am scared that some people will judge me.” – 12-14-year-old student, female, public school.

“In the middle of the pandemic, that was the time when our self-esteem lowered, breakdowns happened so after COVID, we became more shy.” – 15-17-year-old student, female, public school.

Some parents noticed that, as their children went back to school, their social personalities were returning to how they were prior to quarantine restrictions. Children who became more reserved during the pandemic became more outgoing and confident again.

“He has become cheerful… His routine became normal when he returned to face-to-face.” – mother, public school.

“Actually, his confidence went down maybe because he’s not in school, but now that he went back to school, he is slowly getting back to what he is used to be.” – mother, private school.

However, some parents noticed that their once sociable children remained reserved and shy after going back to in-person learning.

“She transformed from being an extrovert to an introvert… She was confined into the house. She doesn’t see any friends. Even her other classmates, she doesn’t see them. So her companion was only her brother.” – mother, private school.

“The self-esteem has changed because the environment has changed. If the classmate, the teacher used to be there, the communication with friends was lost, many were lost. The attitude has actually changed. The type that used to be loud, talkative became shy… When they met someone outside, they will move away [*from*] that person.” – mother, public school.

A couple of stakeholders also described their observations of changes in social behavior after the return to in-person learning. The main observation was that students engaged less in social interaction, choosing instead to use their cell phones or computers rather than hang out with their friends. They also thought that the social distancing restrictions due to the pandemic inhibited development of healthy socialization habits in the students.

“I think that the main concern of the mothers that we talk to is that… now [*their kid*] just doesn’t want to go out. They want to play on their phone or computer. It’s really difficult to encourage their children to go out because they are used to that lifestyle.” – psychologist, NGO employee, female.

“During their growing up years, their interaction was very limited. Now that they are older, they are 3 years older, they have forgotten how to initiate a conversation with me, how to make friends… Since they are very used to online communication, they had a hard time face-to-face because it was very awkward for them. There are those adolescents who seem to be more conscious of how they act, how they behave.” – psychologist, NGO employee, female.

### Recommendations

Students, parents, and stakeholders were also asked about ideas for future interventions that could be implemented to support students as they reacclimate to in-person learning after experiencing major learning and social disruptions during the pandemic. The participants gave recommendations that included curriculum reform, an increase in support services in schools, and an emphasis on extracurricular activities to give students more opportunities to socialize with their peers.

#### Academic support and curriculum restructure

One of the main impacts of remote learning on students was the gap in knowledge that students, caregivers, and stakeholders all noticed after the return to in-person learning. To address this, some students recommended expanding access to tutoring services. Others recommended restructuring the curriculum to allow students who fell behind the opportunity to catch up to their peers and thus, continue schooling on the same level. A few stakeholders similarly emphasized the need to prioritize learning recovery efforts to support students’ academic progress.

“Improve the system and give catch up [*for*] the students just like now they cannot read properly. They should give enough time and effort to catch up for the students who are slow and to avoid discrimination.” – 15-17-year-old student, male, public school.

“Even before pandemic, the level/academic performance of our students is not that high and when the closure happened, it became worse. Because the students’ interaction with their teachers is very limited so it became lower. So there’s a need to focus on the learning recovery of the students.” – city education affairs unit representative, female.

One student suggested continuing the school schedule that had been implemented during remote learning with fewer classes each day and reduced requirements, and one stakeholder agreed.

#### Social and emotional support services

Stakeholders and some older students suggested increasing opportunities for students to socialize with each other by expanding extracurricular activities and including more hands-on activities in the classroom. The thinking behind this suggestion was to lean into students’ interests to get them more engaged with academics and each other, and to reclaim activities that had been taken away by pandemic restrictions.

“In terms of social interaction, that’s because not all people are comfortable with face-to-face interactions. So I think we need to bring back socialization in schools…. When high school and grade school have intramurals, there are dance events. I think it’s something that we have to slowly incorporate in today’s curricula.” – psychologist, NGO employee, female.

Another recommended intervention was to increase support services, allowing students to access school counselors to reflect on experiences during remote learning and receive mental health support.

“More guidance counselors, everyone’s health is suffering no matter what age it is. They encourage [*talking to guidance counselors*], but there is a difficulty in our batch, because the assigned guidance counselor in our batch, their schedule always hits our afternoon classes, although they give a list of the schedule, it is really difficult to skip the class…” – 15-17-year-old student, male, public school.

Stakeholders recognized that students were not the only ones who needed psychosocial support, highlighting the need for coordinated mental health and support for parents and teachers as well, including training, guidance, and accommodations to help families and educators effectively support students’ learning and well-being.

“I think training for students and also for family members is very important through the help of course of mental health professionals… I think the difficulty is for parents to find the time to engage in this kind of training aside from the cost as well. Maybe the support they need… would it be from the schools or from the government? That’s it.” – psychologist, NGO employee, female.

“For teachers, we also provide them with a webinar on understanding for this common goal of supporting the students, to reach it or meet it. For executive functioning for the students, executive functioning for the parents, executive functioning for the teachers. So, we understand nature and then we can provide all the necessary interventions or accommodations for the students.” – guidance counseling unit head, public school, female.

## Discussion

School closures due to the COVID-19 pandemic are shown to have impacted school children globally [1–3, 5]. We conducted a study in Metro Manila, Philippines, where restrictions were particularly stringent, and schools were closed to in-person learning for over two years, resulting in one of the longest COVID-19 induced school closures worldwide [13, 22]. This context offered a unique opportunity to assess the consequences of prolonged school closures in a setting marked by exceptional duration and intensity of restrictions. To capture the breadth of impact, data were collected from multiple groups affected by the closures, including students, parents/caregivers, and key community stakeholders, allowing for a more comprehensive understanding of varied experiences and perspectives. Our data collection occurred about one year after schools fully re-opened to in-person learning allowing individuals to reflect on all their experiences at multiple time points and under different circumstances (abrupt closure, extended remote learning, transition back to in person schooling). The findings reported here contribute to the emerging body of knowledge around the perceived and experienced impacts of remote learning due to COVID-19 among school-aged children as well as their caregivers and teachers [1–3, 25–27].

Overall, we found that primary and secondary school students experienced challenges transitioning from in-person to remote learning, and from remote learning back to in-person learning, due to technological barriers, increases in quantity and difficulty of schoolwork, and changes in engagement with peers and teachers. Students and teachers alike had trouble adjusting to remote learning formats, and students reported feeling less motivated to engage in school activities. Both students and stakeholders noticed learning loss and social setbacks during this time.

### Academic impact

Students in our study faced many challenges with remote learning, including distractions in their learning environment, difficulties adapting to new learning methods, and problems with engagement in their academics. These barriers were not limited to the Philippines – students across the globe reported facing similar conditions. In nearby Thailand, families dealt with multiple children learning remotely, with half of respondents having only one or even no devices in the household. Like in the Philippines, many parents also reported having to help their children with online learning [26]. Even in the Netherlands, which boasts the highest rates of broadband access, an equitable system of school funding, and limited full school closures, primary school students reported receiving inadequate academic instruction at home and exhibited impediments in cognitive and socioemotional development [6, 28].

Consistent with findings from other studies, students in our study reported decreased motivation, difficulty retaining lessons, and concerns about falling academically behind during remote learning. Though we did not quantitatively measure changes in learning levels in our study, many students reported perceived learning loss and academic stakeholders and students’ caregivers also observed substandard reading and writing skills in younger students and lack of foundational knowledge to build upon in older students. Another study conducted in the Philippines among college students had similar findings–students reported that their learning environment at home was the biggest challenge to online learning, and they felt isolated and disinterested in online classes [2]. Other studies found learning loss and decreases in academic performance due to school closures, reflecting the results of perceived learning loss in participants of our study [1, 4, 7, 29]. At ages 8–17 years, our study population are experiencing critical development stages for cognitive and social development, learning to make independent decisions and develop their identity [30, 31]. Thus, it is vital for students in school to be learning not only for academic enrichment, but for their own self-growth [30–32].

### Social-emotional impact

Childhood and adolescent years are also important periods for social development [33, 34]. The isolation from peers and extended time with families at home during pandemic restrictions resulted in both positive and negative outcomes. Some students reported growing closer to their peers and making friends over online games, while others felt disconnected from friends. There are many other studies where students reported feelings of isolation and increased screentime in order to connect with friends [2, 25, 35, 36]. A qualitative study done in Canada with adolescents aged 9 to 12 years and their parents reported that children mentioned lack of socialization as one of the biggest impacts of COVID-19 and noted the value of virtual interactions with peers to alleviate loneliness [35].

Many students appreciated spending more time with their family; however, extended time together in quarantine sometimes exacerbated existing problems. A review looking at how the pandemic affected adolescents and their familial relationships had similar findings, with some studies describing positive interactions, others reporting negative relationships, and still others reporting no change [37]. One key theme across the studies in Campione-Barr’s review was that the family dynamic was very important to child and adolescent mental health [3, 38]. For example, greater family resilience (i.e., maintaining quality relationships with family friends, finding good ways of coping) was associated with both lower maternal and child mental health symptoms in a study from Australia [3]. In contrast, a study in the U.S. found that lower family functioning during the pandemic was associated with loneliness in both middle childhood and adolescence [38]. The effect of increased family time may have been helpful or harmful to the child’s mental health depending on the overall family dynamic.

In our study, setbacks in social development became more apparent after the transition back to in-person learning as students felt uncomfortable socializing with peers, fearing judgment or just feeling too shy to approach each other. These findings highlight the central role of socialization in children and adolescents’ development. During these time periods, children and adolescents are developing their temperaments and social identities through interacting with and identifying with peers [33, 34]. However, after living through the pandemic with limited socialization opportunities, parents and teachers have noted only negative outcomes on social development in students [27, 39]. In one study, elementary school educators note that isolation and limited social interactions was developmentally detrimental for their students [39].

### Lessons learned and recommendations

When asked to identify areas that needed more attention, students in this study mentioned the need for increased academic support and curriculum restructuring to allow them to catch up to their peers and get back on track academically. As learning loss is not a new concept to academia, programs and frameworks have been created to help students catch up after a break in school, including the RAPID framework proposed in 2022 by UNICEF in response to COVID-19 associated learning loss [40]. In particular, the Philippines has implemented the Dynamic Learning Program (DLP), consisting of make-up classes, catch-up sessions, parallel classes, activity-based engagement, student portfolios, and reduced homework policies [41]. Previously implemented in individual settings in the Philippines [42, 43], the DLP was formally implemented by DepEd in November 2024 to help mitigate the impact of class disruptions due to the annual typhoon season, but is relevant in the context of helping students recover from pandemic learning loss as well. Remediation classes held early in the pandemic in the Netherlands to mitigate learning losses that occurred during the first months of lockdown restrictions showed an increase in test scores in those who participated in the classes compared to those who did not. Further analysis found that individual or small-scale programs, especially those with a focus on cognitive skill development (math, reading), had the strongest impact compared to the other types of programs included (larger group sizes, non-cognitive skill development focus) [44]. Models exploring the impact of short-term remediation classes on mitigating learning loss show positive results, but cannot fully make up for the learning loss that occurred [45, 46].

Other mitigation efforts, such as reorientation of instruction (adapting and customizing lessons to students’ learning levels), as suggested by Kaffenberger [45], or broad instruction and curriculum reform as described by Angrist [46], could support improving learning levels among students. However, these efforts at school-level may not be enough. In the Philippines, there was a large migration of both students and teachers from private schools into free public schools, due to tuition costs and low pay. Private schools nationwide had to close after enrollment decreased by 67% in 2022. A consortium of private schools has asked for government assistance to expand the scholarship program to make private schools more accessible to students [47]. Making private school education more accessible will allow students to remain at their school, giving students more stability in their educational journey, though this will require governmental support. Students who stay at their new school will need academic support for a new curriculum as well as social support to transition smoothly into their new environment.

A wider group of participants, including both students and stakeholders, said that students would greatly benefit from social and emotional support programs after the experiences of COVID-19 and the ensuing restrictions. School-sponsored events such as camping, intramural sports, and dances would encourage peer socializing. A study done among students aged 5 to 14 years in Canada explored the importance of the school as a venue of peer-to-peer and student-to-teacher interaction, and the value of going to school in students’ social lives [25].

Another recommended service to help students was making mental health support available in the school. A very small study was done at a school in the Philippines among female adolescents aged 13–16 with depression, evaluating the effectiveness of bibliotherapy (use of the written materials for healing and development) in reducing depression. Participants in the intervention group (six-week program of eight modules) reported lower depression scores across three scales used to evaluate levels of adolescent depression [48].

Stakeholders believed that parents and teachers would also benefit from mental health support as well as training programs around supporting students during school. A study done in a school in the United States looked at the effects of a teacher-administered intervention to reduce anxiety in students aged 5–11 years. The students exposed to the intervention showed greater levels of improvement in anxiety [49], and teachers also benefited from the intervention, showing greater confidence in their knowledge of anxiety reduction techniques [50].

Learnings from the experiences of students, caregivers, and stakeholders during school disruptions due to the COVID-19 pandemic may also give us insight into how these populations may react to future disruptions. Given the vulnerability of the Philippines to natural disasters such as typhoons, and the growing threat of epidemics in general, there is a need for pre-disruption preparedness and post-disruption support and programs to better provide for learning students, planning for academic curriculums and methods of teaching during times of disruption [51], as well as responding to policy updates [52].

### Strengths & limitations

As far as we know, our study is the first qualitative study assessing the impact of COVID-19 pandemic restrictions done in the Philippines to include students, caregivers, and relevant stakeholders. One of the strengths of this study was the setting: the unique situation of having had one of the world’s longest school closures created an opportunity to learn about the impacts of long-term lockdown measures on child and adolescent academic and social development. There is a dearth of research on this topic coming out of Southeast Asia in particular. Additionally, engaging directly with both students and student-facing stakeholders gave us rich insight into the personal experiences of remote learning and the transition back to in-person learning. Interviewing caregivers as well offered another point of view on the student experience and explored intricate home dynamics during the lockdown period. We also interviewed a range of ages among the students, from 2nd -3rd graders to students about to graduate senior high school. Though we did not do deep analysis of the differences between age groups, we could see the differences in impact of changing schooling conditions through the statements that the participants made.

Our study took place almost one year after the students returned to school for in-person learning, which could have introduced recall bias into the participant responses about their experiences during lockdown from over a year prior. However, this timing allowed us to get insight into the transition back to and the experience of adapting to in-person learning. Another limitation was that we did not collect quantitative measures of learning loss or social development which limits our analysis of the impact of the school closures. Nevertheless, the qualitative nature of the participants’ responses grants understanding into the experiences of students and the people involved in the students’ lives.

## Conclusion

School closures due to COVID-19 pandemic quarantine restrictions had a great effect on students’ academic experiences and social skill development in Metro Manila, Philippines. The study’s results reveal that students felt instruction during remote learning was lacking compared to in-person learning, which negatively impacted the transition back to in-person learning after quarantine restrictions were lifted. Students also experienced social setbacks, including challenges in interacting and reconnecting with peers and teachers after prolonged isolation. Participants in our study recommended that academic challenges faced during in-person learning after perceived ineffective remote learning should be remedied using additional academic support in schools and curriculum restructure. Social and mental support services in schools should also be provided to help students, caregivers, and teachers adjust to the lingering effects of the pandemic and their impact on people’s development and wellbeing.

## Supplementary Information

Supplementary Material 1.

Supplementary Material 2.

Supplementary Material 3.

Supplementary Material 4.

Supplementary Material 5.

## Data Availability

The thematic analyses used to inform this manuscript are on Figshare at the following link: https:/figshare.com/articles/dataset/Philippines_students_caregivers_and_stakeholders_thematic_analysis/30456161.
