# Supplementary material for: Behind the screens: perceived impact of COVID-19 on education and the learning environment among school-aged children in the Philippines
Source: BMC Public Health. 2026 Apr 13;26:1653. doi: 10.1186/s12889-026-27305-4 (PMC13196253; doi:10.1186/s12889-026-27305-4)
Supplement: Supplementary file 1 — Supplementary Material 1. [file 12889_2026_27305_MOESM1_ESM.pdf]

Assessing the impact of the COVID-19 pandemic on the health and emotional lives of children and their adult caregivers in Metro Manila, Philippines

FOCUS GROUP DISCUSSION (FGD) GUIDE

STUDENTS – 15-17 Year Olds

FOCUS GROUP DISCUSSION (FGD) GUIDE	
FGD Identification Number	
FGD Facilitator Name	
FGD Note Taker	
Date of FGD	
FGD Site ID Number	
FGD Start Time (HH:MM)	
FGD Stop Time (HH:MM)	
Data Check Performed by	
Data Transcription Date	
Transcribed by	

Before we start, I want to remind you of basic rules which we should all observe:

- We are interested in your personal experiences and opinions. There are no right or wrong answers.
- If there are any questions that you feel uncomfortable answering, you may skip those.
- Everything we talk about is confidential.
- We will use fictitious, not real names throughout the discussion to maintain confidentiality.
- During the focus group discussion, we ask that you not use real names or anything that would identify others.
- We ask each participant to keep what it said in this group to themselves and not gossip about other people's contributions.
- The information gathered will not affect or interfere with your schooling or any other non-academic services that you receive at school.
- The focus group discussion will be recorded to ensure that it is carried out as planned, and to help us in supplementing our written notes and ensuring their accuracy.
- The focus group discussion will last approximately 1.5 hours.

<table>
<tr><th>PRIMARY QUESTIONS</th><th>FOLLOW-UP QUESTIONS (PROBES)</th></tr>
<tr><td colspan="2"></td></tr>
<tr><td colspan="2">ICE BREAKER ACTIVITY
(Start with ice breaker activity)</td></tr>
<tr><td colspan="2"></td></tr>
<tr><td colspan="2">1. EXPERIENCE AND PERSPECTIVES OF COVID-19
First, we would like to talk about COVID-19.</td></tr>
<tr><td>How would you define/describe COVID-19?</td><td>PROBE for
● Contagious illness
● Flu-like and people can feel sick
● Some people do not feel sick
● Can be deadly
● There is a vaccine to help prevent COVID-19
Read formal definition of COVID-19
(COVID-19, an illness caused by the coronavirus, is like the flu and can cause a person's body to feel sick. Most people who have the virus may have a cough, fever, chills (feeling cold) or body aches.)</td></tr>
<tr><td>Where do you get your information about COVID-19 from? Did you feel like you understand what COVID-19 is and have enough information about how it spreads, ways to protect yourself from getting it, how to access vaccinations, etc?</td><td>PROBE for
● TV, radio, friends, family, internet, social media
● Do you trust the information that you are getting about COVID-19? What is a trustworthy source of this information?</td></tr>
<tr><td colspan="2"></td></tr>
<tr><td colspan="2">2. PERSONAL IMPACT OF COVID-19 AND ASSOCIATED RESTRICTIONS
Next, we would like to talk to you about the impact of COVID-19 and the associated restrictions on your lives, specifically. By COVID-19 associated restrictions, we are referring to national lockdowns, travel restrictions, and quarantine/isolation periods. Let’s talk about the effects that you might have experienced at home as well as with friends and then we are going to talk specifically about the school closures.</td></tr>
<tr><td>How did your home life change due to COVID-19-and COVID-19 related restrictions?</td><td>PROBE for
● Interaction with parents
● Interaction with siblings/others in the home
● Changes in people living in household
● Moving/relocating
● Illness/death
● Access to services- health? Other support services?
● Finances (ability to afford/pay for household goods/food)
● Access to food</td></tr>
</table>

PRIMARY QUESTIONS	FOLLOW-UP QUESTIONS (PROBES)
	• Access to parks or recreation facilities
Did you or anyone in your house get sick/fall ill with COVID-19?	• Who got sick?<br>• Did they need to go to the hospital? What was that experience like?<br>• If at home, did they isolate away from others?<br>• What was the experience in the house like?<br>• How did you manage or cope with the situation?<br>• How are those that got COVID-19 feeling now?
How did your relationship with friends change due to COVID-19-and COVID-19 related restrictions?	***PROBE for***<br>• Decrease in amount of time you spent with your friends<br>• How did you stay in touch with friends? Was it easy or hard?<br>• How did your social activities change? Were you able to continue participating in social activities? Did you participate in any new social activities? How? With whom?<br>• Did you experience any changes in romantic relationship? How did you stay in touch with your partner?
When you think about the COVID-19 pandemic and the associated restrictions, what were the main challenges you experienced in your life?	***PROBE for***<br>• Economic/financial<br>• Illness or death (self, family, friends)<br>• Social relationships<br>• Romantic relationships<br>• Lifestyle and wellbeing priorities<br>• Not being at school<br>• Challenges with family<br>• Managing daily living activities<br>• Managing health and wellbeing
When you think about the COVID-19 pandemic and the associated restrictions, what aspects of your life became easier or more enjoyable?	***PROBE for***<br>• Increased sense of community spirit experienced<br>• Break from routine<br>• More free time<br>• More time with family<br>• Nothing
**3. IMPACT OF COVID-19 AND RESTRICTIONS ON SCHOOLING AND EDUCATION**	

PRIMARY QUESTIONS	FOLLOW-UP QUESTIONS (PROBES)
Now we will focus on the impact of COVID-19 and related restrictions on your experience in school and the transition from in-person schooling to remote schooling.	
What was school like for you prior to COVID-19?	*Allow participants to talk about their individual experiences and see what common themes emerge.*<br>***PROBE for***<br>● Amount of time in school<br>● Travel to school<br>● Schooling hours<br>● Number of students (total school population and class sizes)<br>● Interaction with other students<br>● Interaction with teachers<br>● Materials needed<br>● Types of assignments<br>● Quality of education / teaching<br>● Benefits of school?<br>● Challenges related to school?
Please describe when and how your school closed at the beginning of the COVID-19-related restrictions.	***PROBE for***<br>● When (month/year) did your school close?<br>● How were you notified? By whom?<br>● How quick was the transition?<br>● Who supported you during the transition?
How did the experience of “going to school” change after in-person school closed?	***PROBE for***<br>● Amount of time ‘in school’<br>● Method of education (remote/virtual, no schooling, home schooled, etc.)<br>● Schooling hours<br>● Number of students (total school population and class sizes)<br>● Interaction with other students<br>● Interaction with teachers<br>● Materials needed<br>● Types of assignments<br>● Quality of education / teaching
What were some of the challenges you experienced with transitioning to remote/virtual/at home schooling?	***PROBE for***<br>● Access to materials needed for schoolwork<br>● Ability to focus on schoolwork<br>● Interest in school<br>● Different ways of teaching and learning

PRIMARY QUESTIONS	FOLLOW-UP QUESTIONS (PROBES)
	● Support received at home to complete your schoolwork/learning activities
Did you have all of the materials (paper/pens, gadgets, etc.) and resources (internet connection, TV broadcast, etc.) that you needed to make the transition to remote/virtual/at home schooling?	***PROBE for***<br>● Please explain.<br>● How did you get the materials?<br>● Did your family have to spend money on any materials or resources?<br>● Did the school or another organization provide any materials?<br>● Did you have to share the materials or resources with any other household members (siblings, caregivers, etc.)?
During the time your school was closed, did anyone from your school ever come to your home to check in on you and how your schoolwork was going?	***PROBE for***<br>● If yes, who came to your house?<br>o What did they do during their visit?<br>o Did they show you how to use any new materials or gadgets needed for school?<br>o Was the visit helpful?<br>● If no, was someone from school supposed to visit you at home?
Who helped you at home with your remote/virtual/at home schooling?	***PROBE for***<br>● Parents/caregiver<br>● Siblings<br>● Teacher<br>● Other students / peers<br>● Others<br>How did they help you?<br>Did you get enough support at home to complete your schoolwork?
How did your academic performance change after in-person schooling closed?	***PROBE for***<br>● Why do you think your academic performance increased, decreased or stayed the same?<br>● Do you feel that you learned more or less in the remote/virtual/at home school environment compared to in-person?
Aside from your academic classes, did you take part in other types of enrichment and/or support programs at school prior to COVID-19? How did the programs and/or	***PROBE for***<br>● Pre/post school programs<br>● Nutrition/feeding programs<br>● Guidance counselling

PRIMARY QUESTIONS	FOLLOW-UP QUESTIONS (PROBES)
your participation change at the beginning of the COVID-19 school closures?	● Academic tutoring (subject-specific or general)<br>● Coaching for academic competitions<br>● School trips/field trips<br>● Sports<br>● Arts<br>● Others
Please describe how your new remote/virtual/at home schooling experience changed over time.	***PROBE for***<br>● Changes in teaching methods used<br>● Changes in technology utilized<br>● Changes in hours<br>● Changes in types of assignments<br>● Changes in organization and structure<br>● Changes in quality of education<br>● Overtime, did it improve overall or not?
What positive experiences did you have during the school closures?	***PROBE for***<br>● New activities<br>● More time with family<br>● More time for other activities<br>● More time to study<br>● More time to play<br>● More time to watch TV, movies, etc.
We all experience different emotions and feelings throughout our lives and when major events happen, such as your school closing due to COVID-19, it is normal to experience many different emotions and reactions that may impact your mood and physical health. We are interested to know how the school closures impacted your well-being, both emotionally and physically.	
How did your mood and emotions change after your **school closed** due to COVID-19?	***PROBE for***<br>● Changes in any of the following emotions:<br>o Happiness<br>o Sadness<br>o Fear<br>o Anger<br>o Anxiety<br>o Loneliness<br>● How did you cope with/manage these changes in your mood and emotions?<br>o Starting/participating in a new hobby/activity<br>o Engaging in outdoor activities

PRIMARY QUESTIONS	FOLLOW-UP QUESTIONS (PROBES)
	o Engaging in online activities (social media, gaming, virtual social activities)<br>o Self-care (taking time for yourself, listening to music, reading a book, writing in a journal, meditating, praying, etc.)<br>o Support from family<br>o Support from friends<br>o Other
How did your physical health change after your **school closed** due to COVID-19?	***PROBE for***<br>• Changes in physical exercise<br>• Changes in nutrition/eating habits<br>• Changes in drinking habits<br>• Changes in sleep<br>• Exposure to illnesses/getting sick<br>• Feeling healthy or not
**4. TRANSITION BACK TO SCHOOL (reopening)**<br>*Now we want to hear about your experience transitioning back to in-person schooling after the school closures ended.*	
Please describe your transition back to school.	***PROBE for***<br>• When did your school reopen to in-person schooling?<br>• How long were you away from in-person learning?<br>• When were you told you would return to school?<br>• How quick was the transition back?<br>• COVID-19 safety measures<br>• Hybrid vs. Fully in-person?
How did you feel about going back to school in person?	***PROBE for***<br>• Excited/happy<br>• Anxious/Nervous<br>• Fearful<br>• Sad<br>• Mixed<br>***Why?***
Is there anything that you would have changed about your transition back to school?	***PROBE for***<br>• New school schedule and methods of teaching (In-person, hybrid, non-hybrid)<br>• Slower or quicker transition back<br>• Vaccination requirements

PRIMARY QUESTIONS	FOLLOW-UP QUESTIONS (PROBES)
	• COVID-19 precautions in schools (masks, temperature checks, hand sanitizer stations, etc.)<br>• Travel/transport going to school and back
Since you have returned to in-person school, have you noticed a difference in the quality of in-person teaching and your educational experience compared to remote/virtual/at home schooling?	***PROBE for***<br>• Quality of teaching/teachers<br>• Quality of classes<br>• Quality of assignments<br>• Why do you think this is?
Again, we would like to hear from you about the different emotions and feelings you experienced related to school closures, in this case the reopening and transition back to in-person schooling.	
How has your mood and emotions changed after the transition **back to in-person schooling**?	***PROBE for***<br>• Changes in any of the following emotions:<br>o Happiness<br>o Sadness<br>o Fear<br>o Anger<br>o Anxiety<br>• How are you coping with/managing the changes in your mood and emotions?
How has your physical health changed after the transition **back to in-person schooling**?<br><br>How was your lifestyle changed after the transition back to in-person schooling?	***PROBE for***<br>• Changes in physical exercise<br>• Changes in nutrition/eating habits<br>• Changes in drinking habits<br>• Changes in sleep<br>• Exposure to illnesses/getting sick<br>• Feeling healthy or not<br>• COVID-19 safety precautions
**5. IMPROVEMENT IDEAS**<br>*Now we are interested in hearing your suggestions for how the transitions from in-person schooling to remote/virtual/at home schooling and then back to in-person school could have been improved.*	
How could the transition from **<u>in-person schooling to remote/virtual/at home schooling</u>** (at the beginning of COVID-19) have been better?	***PROBE for***<br>• For you<br>• For your family<br>• Slower/faster transition<br>• Ensure all students have materials they need for remote/virtual/at home schooling<br>• Support from school<br>• Support from teachers

PRIMARY QUESTIONS	FOLLOW-UP QUESTIONS (PROBES)
How could the transition **back to in-person schooling** have been better?	***PROBE for***<br>● For you<br>● For your family<br>● Slower/faster transition<br>● Ensure all students have materials they need for in-person school<br>● Support from school<br>● Support from teachers
How do you think the changes in your schooling due to COVID-19 will impact your future?	***PROBE for***<br>● Academic achievements<br>● Mental health<br>● Physical health<br>● Social health
What emotional, academic, and/or social support would help address the continued impact of COVID-19 pandemic and the associated school closures on your life?	***PROBE for***<br>● Emotional support from caregivers<br>● Emotional support from friends<br>● Access to guidance counsellors who are well-trained to support students<br>● Academic support from caregivers<br>● Access to and support from teachers<br>● Materials/technology needed to complete academic work<br>● More opportunities to socialize with friends/other students<br>● Less social opportunities (to avoid social anxiety)
We are at the end of the discussion. Do any of you have anything to add? Or is there anything you forgot to tell us?	
