# Supplementary material for: Behind the screens: perceived impact of COVID-19 on education and the learning environment among school-aged children in the Philippines
Source: BMC Public Health. 2026 Apr 13;26:1653. doi: 10.1186/s12889-026-27305-4 (PMC13196253; doi:10.1186/s12889-026-27305-4)
Supplement: Supplementary file 2 — Supplementary Material 2. [file 12889_2026_27305_MOESM2_ESM.pdf]

Assessing the impact of the COVID-19 pandemic on the health and emotional lives of children and their adult caregivers in Metro Manila, Philippines

IN-DEPTH INTERVIEW GUIDE

KEY STAKEHOLDERS

IN-DEPTH INTERVIEW GUIDE WITH KEY STAKEHOLDERS	
Participant ID Number	
Participant Gender	
Interviewer Name	
Date of Interview	
Interview Start Time (HH:MM)	
Interview Stop Time (HH:MM)	
Data Check Performed by	
Data Transcription Date	
Transcribed by	

Before we start, I want to remind you of three things:
- First, we are interested in your personal experiences and opinions. There are no right or wrong answers.
- Second, if there are any questions that you feel uncomfortable answering, we can skip those.
- And third, everything we talk about is confidential. The information gathered will not be shared with anyone and will not influence or interfere with your child's schooling or any other non-academic services that your child receives at school.

The interview will be audio-recorded to assure that it is carried out as planned, and to help us in supplementing our written notes and ensuring their accuracy.

The interview will last approximately 1.5 hours.

INTERVIEWER INSTRUCTIONS
- BE SURE TO ASK EACH MAIN QUESTION.
- THE FOLLOW-UP QUESTIONS ARE TO HELP YOU OBTAIN MORE INFORMATION FROM THE PARTICIPANT AND MAY ENCOURAGE THE PARTICIPANT TO TALK.

- ALL OF THE QUESTIONS IN THE COLUMN LABELED 'FOLLOW-UP QUESTIONS' ARE TO BE ASKED IF THE PARTICIPANT DOES NOT ADDRESS THEM WHEN ANSWERING THE PRIMARY QUESTION.

	Question	Probes & Instructions
**1. PARTICIPANT CHARACTERISTICS**<br>*First, we would like to ask you some questions about yourself and your work.*		
	What gender do you identify with?	
	What is your age?	
	What type of organization do you work for?	
	How long have you worked with this organization? (years/months)	
	Can you briefly tell me about your role at this organization and how you work with/interact with schools, the education system, school-aged children, their caregivers, etc.	***PROBE for***<br>1. *Types of stakeholders:* educators (i.e., principals and teachers) from both public and private schools, service providers (i.e., psychologists, psychiatrists, pediatricians), researchers, child health and child mental health advocates and policy makers, including representatives from the Departments of Health and Education<br>2. Private vs. Public schools<br>3. Interaction with Departments of Health and Education
**2. COVID-19 POLICIES – GENERAL**<br>*Next, we would like to talk to you about COVID-19 and the associated restrictions. By COVID-19 associated restrictions, we are referring to national lockdowns, travel restrictions, and quarantine/isolation periods. Later we are going to talk specifically about the school closures.*		
	Please describe the first COVID-19 associated restrictions and policies put into place in the Philippines.	***PROBE for:***<br>● What- curfew, lockdown, travel, schools, businesses, clinics/hospitals<br>● When- approximate timeline<br>● How- who enforced them, how were they regulated, were they followed
	From your perspective, what was the impact of these policies on the general population?	***PROBE for:***<br>● Community wellbeing

	Question	Probes & Instructions
		• Mental health<br>• Physical health<br>• Economic/financial<br>• Access to resources (health, food, parks, recreation facilities)
	How did these policies impact you and your work?	***PROBE for:***<br>• Were you involved in the implementation of any of these policies? How?<br>• What adjustments did you adopt to continue with your work under these new policies?
**3. IMPACT OF COVID-19 SCHOOL CLOSURES**<br>*Now we will focus on the impact of COVID-19 and related restrictions on students, their caregivers, and teachers.*		
	Please describe the COVID-19 associated restrictions specifically related to school closures.	***PROBE for:***<br>• What- did all schools close? Different policies for private vs. public schools? Did students immediately move to remote learning?<br>• When- approximate timeline<br>• How- who enforced them, how were they regulated, were they followed
	**For policy makers:**<br>How were the decisions around school closures made?	• How were you involved in the decisions to close the schools?<br>• Were you involved in implementing the school closures? How?<br>• What factors informed the decisions and policies around school closures?
	**For non-policy makers:**<br>How did the school closures impact you and your work?	***PROBE for:***<br>• Did your workload increase or decrease?<br>**For teachers:**<br>Were you provided with the materials needed to complete your work?<br>Were you trained on new learning methods?
	From your perspective, what was the impact of these school closures on students?	***PROBE for:***<br>• Negative impacts / challenges<br>• Positive impacts

	Question	Probes & Instructions
		• Social interactions and relationships<br>• Mental health<br>• Physical health<br>• Family/home life<br>• Academic achievements/challenges<br>• Finances/economic
	Are there differences in the impact of the closures on different age groups of students (ages 8-11, 12-14, 15-17)? Please explain.	***PROBE for:***<br>• For ages 8-11<br>• For ages 12-14<br>• For ages 15-17
	From your perspective, what was the impact of these closures on caregivers of school-aged children?	***PROBE for:***<br>• Negative impacts / challenges<br>• Positive impacts<br>• Social interactions and relationships<br>• Mental health<br>• Physical health<br>• Family/home life<br>• Finances/economic<br>• Work/Employment<br>• Time spent helping child with schoolwork
	From your perspective, what was the impact of these closures on teachers?	***PROBE for:***<br>• Negative impacts / challenges<br>• Positive impacts<br>• Social interactions and relationships<br>• Mental health<br>• Physical health<br>• Family/home life<br>• Finances/economic<br>• Work/Employment
**4. REOPENING OF SCHOOLS**<br>*Now we want to hear about the transition back to in-person schooling after the school closures ended.*		
	Please describe the process of reopening the schools and the transition back to in-person schooling.	***PROBE for:***<br>• What- did all schools reopen?<br>• Was there a difference between private and public schools?<br>• When- approximate timeline

	Question	Probes & Instructions
		• How- who decided to reopen the schools? What was the process for reopening? Are all students back at school?
	**For policy makers:**<br>How were the decisions around re-opening of in-person schooling made?	• How were you involved in the decisions to reopen the schools?<br>• Were you involved in implementing the school reopening? How?<br>• What factors informed the decisions and policies around school reopening?
	**For non-policy makers:**<br>How did the school reopening impact you and your work?	***PROBE for:***<br>• Did your workload increase or decrease?<br>**For teachers:**<br>Were you provided with the materials needed to complete your work?<br>Were you trained on new learning methods?
	From your perspective, what has the impact of the reopening of schools been on students?	***PROBE for:***<br>• Negative impacts / challenges<br>• Positive impacts<br>• Social interactions and relationships<br>• Mental health<br>• Physical health<br>• Family/home life<br>• Academic achievements/challenges<br>• Finances
	Are there differences in the impact of the reopening of schools on different age groups of students (ages 8-11, 12-14, 15-17)? Please explain.	***PROBE for:***<br>• For ages 8-11<br>• For ages 12-14<br>• For ages 15-17
	From your perspective, what was the impact of the reopening of schools on caregivers of school-aged children?	***PROBE for:***<br>• Negative impacts / challenges<br>• Positive impacts<br>• Social interactions and relationships<br>• Mental health<br>• Physical health<br>• Family/home life

	Question	Probes & Instructions
		• Finances / work<br>• Time spent helping child with schoolwork
	From your perspective, what was the impact of the reopening of schools on teachers?	***PROBE for:***<br>• Negative impacts / challenges<br>• Positive impacts<br>• Social interactions and relationships<br>• Mental health<br>• Physical health<br>• Family/home life<br>• Finances / work
**5. PEDIATRIC POPULATION NEEDS**<br>*Lastly, we would like to hear about your thoughts on how the impact of the school closures and reopening of schools can be addressed for the pediatric/adolescent population in the Philippines.*		
	In your opinion, what are the greatest needs of school-aged children and adolescents in the Philippines today?	***PROBE for:***<br>• Social interactions / relationships<br>• Mental health<br>• Physical health<br>• Education
	What emotional, academic, and/or social support would help address the continued impact of COVID-19 pandemic and the associated school closures on students?	***PROBE for***<br>• Emotional support from caregivers/family<br>• Emotional support from friends<br>• Access to guidance counsellors who are well-trained to support students<br>• Academic support from caregivers<br>• Access to and academic support from teachers<br>• Materials/technology needed to complete academic work<br>• More opportunities to socialize with friends/other students<br>• Less social opportunities (to avoid social anxiety)<br>• Support from the healthcare system<br>• Community-based support

	Question	Probes & Instructions
	How should this support be provided to students?	***PROBE for:***<br>● Where?<br>● How?<br>● By whom?<br>● What organizations?
	What are the barriers to addressing the needs of students?	***PROBE for:***<br>● Costs<br>● Access<br>● Engagement
	What would facilitate addressing the needs of students?	***PROBE for:***<br>● Funding<br>● Programs<br>● Outreach
	What emotional, work, and/or social support would help address the continued impact of COVID-19 pandemic and the associated school closures on caregivers of school-aged children?	***PROBE for***<br>● Emotional support from spouse/partner<br>● Emotional support from friends<br>● Academic support for child(ren) from school<br>● Child(ren) provided with the materials/technology needed to complete academic work<br>● Flexible work schedules (hours/location)<br>● More employment opportunities<br>● More opportunities to socialize with friends/family
	How should this support be provided to caregivers?	***PROBE for:***<br>● Where?<br>● How?<br>● By whom?<br>● What organizations?
	What are the barriers to addressing the needs of caregivers?	***PROBE for:***<br>● Costs<br>● Access<br>● Engagement
	What would facilitate addressing the needs of caregivers?	***PROBE for:***<br>● Funding<br>● Programs<br>● Outreach
	What emotional, work, and/or social support would help address the continued impact of COVID-19 pandemic and the associated school closures on teachers?	***PROBE for***<br>● Emotional support from school

	Question	Probes & Instructions
		• Emotional support from spouse/partner<br>• Emotional support from friends<br>• Additional educational training<br>• Additional training to learn how to support students who may be struggling academically and/or emotionally after the transition back to school<br>• Additional training on new learning technology<br>• Work/life balance<br>• More opportunities to socialize with friends/family
	How should this support be provided to teachers?	***PROBE for:***<br>• Where?<br>• How?<br>• By whom?<br>• What organizations?
	What are the barriers to addressing the needs of teachers?	***PROBE for:***<br>• Costs<br>• Access<br>• Engagement
	What would facilitate addressing the needs of teachers?	***PROBE for:***<br>• Funding<br>• Programs<br>• Outreach
	Anything else to add that we have not covered?	

Thank you so much for participating in this interview.
