# Supplementary material for: Behind the screens: perceived impact of COVID-19 on education and the learning environment among school-aged children in the Philippines
Source: BMC Public Health. 2026 Apr 13;26:1653. doi: 10.1186/s12889-026-27305-4 (PMC13196253; doi:10.1186/s12889-026-27305-4)
Supplement: Supplementary file 3 — Supplementary Material 3. [file 12889_2026_27305_MOESM3_ESM.pdf]

Assessing the impact of the COVID-19 pandemic on the health and emotional lives of children and their adult caregivers in Metro Manila, Philippines

IN-DEPTH INTERVIEW GUIDE

8-11 YEAR OLD STUDENTS & THEIR CAREGIVERS

IN-DEPTH INTERVIEW (IDI) GUIDE	
IDI Identification Number	
Student Participant ID Number	
Caregiver Participant ID Number	
IDI Facilitator Name	
IDI Note Taker	
Date of Interview	
IDI Site ID Number	
IDI Start Time (HH:MM)	
IDI Stop Time (HH:MM)	
Data Check Performed by	
Data Transcription Date	
Transcribed by	

Before we start, I want to remind you of three things:
- First, we are interested in your personal experiences and opinions. There are no right or wrong answers.
- Second, if there are any questions that you feel uncomfortable answering, we can skip those.
- And third, everything we talk about is confidential. The information gathered will not be shared with anyone and will not influence or interfere with your or your child's schooling or any other non-academic services that you or your child receives at school.

The interview will be audio-recorded to assure that it is carried out as planned, and to help us in supplementing our written notes and ensuring their accuracy.

The interview will last approximately 1 hour.

INTERVIEWER INSTRUCTIONS
- BE SURE TO ASK EACH MAIN QUESTION.
- THE FOLLOW-UP QUESTIONS ARE TO HELP YOU OBTAIN MORE INFORMATION FROM THE PARTICIPANT AND MAY ENCOURAGE THE PARTICIPANT TO TALK.

- ALL OF THE QUESTIONS IN THE COLUMN LABELED 'FOLLOW-UP QUESTIONS' ARE TO BE ASKED IF THE PARTICIPANT DOES NOT ADDRESS THEM WHEN ANSWERING THE PRIMARY QUESTION.

<table>
<tr><th>PRIMARY QUESTIONS</th><th>FOLLOW-UP QUESTIONS (PROBES)</th></tr>
<tr><td colspan="2"></td></tr>
<tr><td colspan="2">1. GENERAL IMPACT OF COVID-19
First, we would like to talk to you about the impact of COVID-19 and the associated restrictions on your lives, specifically. By COVID-19 associated restrictions, we are referring to national lockdowns, travel restrictions, and quarantine/isolation periods. Let's talk about the effects that you might have experienced in your life in general—we will talk specifically about the school closures later on.</td></tr>
<tr><td>Both the child and caregiver: How did your life change due to COVID-19-and COVID-19 related restrictions?</td><td>PROBE for
• What was good?
• What was difficult?</td></tr>
<tr><td>Caregiver only:
Did you or anyone in your house get sick/fall ill with COVID-19?</td><td>• Who got sick?
• Did they need to go to the hospital? What was that experience like?
• If at home, did they isolate away from others?
• What was the experience in the house like?
• How did you manage or cope with the situation?
• How are those that got COVID-19 feeling now?</td></tr>
<tr><td colspan="2"></td></tr>
<tr><td colspan="2">2. IMPACT OF COVID-19 AND RESTRICTIONS ON CHILDREN'S SCHOOLING AND EDUCATION
Address the child: Now we want to learn more about how COVID-19 impacted your experience in school.</td></tr>
<tr><td colspan="2">Child only (caregiver support):
<u>Creative activity:</u>
• Ask the child to draw a picture of what their classroom or school looked like before COVID-19 (before the school closures) OR
• Ask the child to choose which images best represent their experience at school before COVID-19 (before school closures)
[Ask the caregiver to work with the child in this activity]</td></tr>
<tr><td colspan="2"><u>Creative activity, introductory question:</u>
<u>Interviewer</u>: Refer to the child's pre-COVID-19 drawing of their classroom/school or selected image
• Could you tell me about your drawing or chosen picture?
• What was school like for you before COVID-19? What were your school activities like?
• Describe a typical day at school.</td></tr>
</table>

PRIMARY QUESTIONS	FOLLOW-UP QUESTIONS (PROBES)
Probe for details about what is included in the child's drawing or selected image. Time spent in school, interaction with other students/friends, interaction with teachers, types of schooling and education.	
**Caregiver only:**<br>Based on what your child described, do you have anything to add about what school was like for your child prior to COVID-19?	***PROBE for***<br>● Amount of time in school<br>● Travel to school<br>● Schooling hours<br>● Number of students (total and class size)<br>● Interaction with other students<br>● Interaction with teachers<br>● Materials needed<br>● Types of assignments<br>● Quality of education / teaching<br>● Benefits of school?<br>● Challenges related to school?
**Caregiver only:**<br>Please describe when and how your child's school closed at the beginning of the COVID-19 related restrictions.	***PROBE for***<br>● When (month/year) did your child's school close?<br>● How were you notified? By whom?<br>● How quick was the transition?<br>● Who supported your child during the transition?<br>● How did your child's new remote schooling experience change over time?
**Child only:**<br>What was school like after you couldn't go to school in-person anymore?<br>*Allow child to answer probes.*<br><br>**Caregiver only:**<br>Do you have anything to add to what your child said about their schooling experience after in-person school closed?	***PROBE for***<br>● Amount of time 'in school'<br>● Method of education (remote/virtual, no schooling, home schooled, etc.)<br>● Schooling hours<br>● Number of students (total school size and class sizes)<br>● Spending time with other students<br>● Spending time with teachers<br>● Materials needed for school<br>● Types of school assignments<br>● Quality of education / teaching
**Child only:**<br>For you, what were some difficult things about not being able to go to school in-person anymore?<br>*Allow child to answer probes.*<br><br>**Caregiver only:**	***PROBE for***<br>● Access to materials needed for schoolwork<br>● Ability to focus on schoolwork<br>● Interest in school<br>● Different ways of teaching and learning

PRIMARY QUESTIONS	FOLLOW-UP QUESTIONS (PROBES)
Do you have anything to add to what your child said about their challenges transitioning to remote schooling?	• Support received at home to complete your schoolwork/learning activities
**Caregiver only:**<br>What were some of the challenges **you experienced** due to your child transitioning to remote schooling?	***PROBE for***<br>• Change in routine<br>• Time spent supporting child with school assignments and technology<br>• Access to materials needed<br>• Ability to focus<br>• Quality of education / teaching
**Child only:**<br>Did you have all the materials (paper/pens, gadgets, etc.) and resources (internet connection, TV broadcast, etc.) you needed to do your schooling at home?<br>*Allow child to answer probes.*<br><br>**Caregiver only:**<br>Do you have anything to add to what your child said about the materials and resources needed to make the transition to remote school?	***PROBE for***<br>• Please explain.<br>• How did you get the materials?<br>• Did your family have to spend money on any materials or resources?<br>• Did the school or another organization provide any materials?<br>• Did you have to share the materials or resources with any other household members (siblings, caregivers, etc.)?
**Child only:**<br>During the time your school was closed, did anyone from your school ever come to your home to check in on you and how your schoolwork was going?<br>*Allow child to answer probes.*<br><br>**Caregiver only:**<br>Do you have anything to add to what your child said about any home visits from the school while the school was closed?	***PROBE for***<br>• If yes, who came to your house?<br>o What did they do during their visit?<br>o Did they show you how to use any new materials or gadgets needed for school?<br>o Was the visit helpful?<br>• If no, was someone from school supposed to visit you at home?
**Child only:**<br>How did your caregiver support you with your schoolwork at home?<br>*Allow child to answer probes.*<br><br>**Caregiver only:**<br>How did you support your child at home with their remote schooling? How?<br>How comfortable were you providing this support?	***PROBE for***<br>• Helping with schoolwork (homework/modules)<br>• Helping with technology for remote/virtual schooling<br>• Helping you to stay focused<br>• Explaining/describing schoolwork<br>Did other people (siblings, other family members, friends) also provide support to you?<br>Did you get enough support at home to complete your schoolwork?
**Caregiver only:**	***PROBE for***

<table>
<tr><th>PRIMARY QUESTIONS</th><th>FOLLOW-UP QUESTIONS (PROBES)</th></tr>
<tr><td>Aside from your child’s academic classes, did your child take part in other types of enrichment and/or support programs at school prior to COVID-19? How did the programs and/or your child’s participation change at the beginning of the COVID-19 school closures?</td><td>• Pre/post school programs
• Nutrition/feeding programs
• Guidance counselling
• Academic tutoring (subject-specific or general)
• Coaching for academic competitions
• School trips/field trips
• Sports
• Arts
• Others</td></tr>
<tr><td>Caregiver only:
Did you take part in any school activities as your child’s caregiver prior to COVID-19? How did your participation / involvement change at the beginning of the COVID-19 school closures?</td><td>PROBE for
• Parent-Teacher Association (PTA)
• Attending or volunteering at school programs (plays, fairs, competitions, etc.)
• Supporting the feeding program
• Other activities</td></tr>
<tr><td>Child only:
What good things happened during the time you couldn’t go to school in-person?
Allow child to answer probes.

Caregiver only:
What positive experiences did you have during the school closures?
Also use the probes for the caregiver</td><td>PROBE for
• New activities
• More time with family
• More time for other activities
• More time to study/do schoolwork
• More time to play
• More time to watch TV, movies, etc.</td></tr>
<tr><td colspan="2">Thank you both for your contributions so far. We have asked about the impact of the school closures on you and your child’s life and now we would like to focus on the impact on emotional and physical health and well-being specifically.
Address the child: We all experience different emotions and feelings throughout our lives and when major events happen, such as your school closing due to COVID-19, it is normal to experience lots of feelings and reactions that may change your mood and physical health. We are going to do another exercise and ask you to name some emotions.</td></tr>
<tr><td colspan="2">Child only (caregiver support):
<u>Creative activity:</u>
• Show the child the different emotion images (emojis) and ask them to name each emotion (happy, sad, fearful, angry, lonely, stressed, anxious). After naming, ask them to describe that emotion and when someone may feel that emotion.</td></tr>
<tr><td colspan="2"><u>Creative activity, introductory question:</u>
<u>Interviewer:</u> Now I am going to describe a situation and you can tell me based on the emojis, how that situation made you feel.
• When you first hear about COVID-19
• Your school closing down because of COVID-19
• Not being allowed to go outside of your home
• Spending more time with your family at home</td></tr>
</table>

<table>
<tr><th>PRIMARY QUESTIONS</th><th>FOLLOW-UP QUESTIONS (PROBES)</th></tr>
<tr><td colspan="2">

- Not seeing your friends (other students) at school during closures
- The amount of school assignments

Probe for why they selected that emotion to describe that situation. If they select multiple emotions, ask them why and to explain.

</td></tr>
<tr><td>

Caregiver only:
Do you have anything to add based on the emotions your child described?

</td><td></td></tr>
<tr><td colspan="2"></td></tr>
<tr><td colspan="2">

4. TRANSITION BACK TO SCHOOL (reopening)
Address the child: *Now we want to hear about your experience with going back to in-person schooling after the school closures ended.*

</td></tr>
<tr><td colspan="2">

Child only (caregiver support):

<u>Creative activity:</u> *Next, I will read a short story.*

SHORT STORY: *Jacob is 9 years old and has been doing schoolwork at home (online/virtual or through modular learning) since the start of COVID-19 when his school was closed. Jacob had a hard time when homeschooling first started as he had never done this kind of schooling before. After a few months, he got used to it and started to enjoy it and participate more than he did when school was in-person. He also liked being at home to do his schoolwork because he was able to spend time with his family and play outside more than before. His mom helped him a lot with his schoolwork.*

Jacob's aunt got COVID-19 and was very sick for a while. She is back home recovering but it was a very scary experience and Jacob and his family are very careful as they do not want to get COVID-19. They do not see other people outside of their family who lives in the same home. Jacob misses his friends, but he understands that distance will keep him and his family safe.

About a week ago, Jacob's school said that the school was reopening for in-person schooling, and students were to come back to school at the end of the month. Jacob felt many different emotions—he was excited to go back to school because he missed his friends and his teachers. He was also nervous because he was now used to the new at home schooling. He was not sure if the schoolwork would be harder now that all the students and teachers were back at school in-person. Jacob was also anxious to return to school because he and his family were not seeing people outside of their family. Jacob was worried he would get COVID-19 at school and then come home and get his family sick. Jacob is overwhelmed by this transition back to school.

- Request the child to reflect on this story.

</td></tr>
<tr><td colspan="2">

<u>Creative activity, introductory question:</u>

<u>Interviewer:</u>

- How does this story make you feel? Are you happy for Jacob? Are you worried about Jacob?
- Do you relate to Jacob at all? How so?
- Do you feel different than Jacob? How so?

</td></tr>
</table>

<table>
<tr><th>PRIMARY QUESTIONS</th><th>FOLLOW-UP QUESTIONS (PROBES)</th></tr>
<tr><td colspan="2">● What was the transition back to school like for you?
● What does your school look like now?
● How is your school the same as it was before COVID-19?
● How is your school different than it was before COVID-19?
● Does your school have any COVID-19 safety precautions in place?

Probe for time spent in school, interaction with other students/friends, interaction with teachers, types of schooling and education.</td></tr>
<tr><td>Caregiver only:
Do you have anything to add to what your child described about their transition back to school?</td><td>If yes, PROBE for
● When did your child's school reopen to in-person schooling?
● How long was your child away from in-person learning?
● When were you told the school was reopening for in-person schooling? How quick was the transition back?
● COVID-19 safety measures
● Hybrid vs. Fully in-person?</td></tr>
<tr><td>Caregiver only:
How did you feel about your child going back to school in person?</td><td>PROBE for
● Excited/happy
● Anxious
● Fearful
● Sad
Why?</td></tr>
<tr><td>Caregiver only:
Is there anything that you would have changed about the transition back to in-person schooling?</td><td>PROBE for
● New school schedule and methods of teaching (In-person, hybrid, non-hybrid)
● Slower or quicker transition back
● Vaccination requirements
● COVID-19 precautions in schools (masks, temperature checks, hand sanitizer stations, etc.)
● Travel/transport going to school and back</td></tr>
<tr><td>Caregiver only:
Since your child has returned to in-person school, have you noticed a difference in the quality of in-person teaching and their educational experience compared to remote schooling?</td><td>PROBE for
● Quality of teaching
● Quality of assignments
● Why do you think this is?</td></tr>
<tr><td colspan="2">Address the child: Again, we would like to hear from you about the different emotions and feelings you (referring to child) / your child (referring to caregiver) experienced related to school closures, in this case the reopening and transition back to in-person schooling.</td></tr>
<tr><td colspan="2">Child only (caregiver support):</td></tr>
</table>

PRIMARY QUESTIONS	FOLLOW-UP QUESTIONS (PROBES)
**Creative activity:**<br>● Remind the child about the different emojis and that you will be asking new questions about them	
**Creative activity, introductory question:**<br>Interviewer: *Using the same emojis from before I am going to describe a situation and you can tell me based on the emojis, how that situation made you feel.*<br>● When your school reopened<br>● When you saw your best friends/buddies back at school<br>● When you saw school "bullies" back at school<br>● When you saw your teacher again<br>● The commute to school<br>● The amount of time spent at school<br>● The amount of school assignments<br><br>*Probe for why they selected that emotion to describe that situation. If they select multiple emotions, ask them why and to explain.*	
**Caregiver only:**<br>Do you have anything to add based on the emotions your child described?	
**Both the child and caregiver:** We are at the end of the interview. Do either of you have anything to add? Or is there anything you forgot to tell us?	
