# Supplementary material for: Behind the screens: perceived impact of COVID-19 on education and the learning environment among school-aged children in the Philippines
Source: BMC Public Health. 2026 Apr 13;26:1653. doi: 10.1186/s12889-026-27305-4 (PMC13196253; doi:10.1186/s12889-026-27305-4)
Supplement: Supplementary file 4 — Supplementary Material 4. [file 12889_2026_27305_MOESM4_ESM.pdf]

Assessing the impact of the COVID-19 pandemic on the health and emotional lives of children and their adult caregivers in Metro Manila, Philippines

FOCUS GROUP DISCUSSION (FGD) GUIDE

CAREGIVERS

FOCUS GROUP DISCUSSION (FGD) GUIDE	
FGD Identification Number	
FGD Facilitator Name	
FGD Note Taker	
Date of FGD	
FGD Site ID Number	
FGD Start Time (HH:MM)	
FGD Stop Time (HH:MM)	
Data Check Performed by	
Data Transcription Date	
Transcribed by	

Before we start, I want to remind you of basic rules which we should all observe:

- We are interested in your personal experiences and opinions. There are no right or wrong answers.
- If there are any questions that you feel uncomfortable answering, you may skip those.
- Everything we talk about is confidential.
- We will use fictitious, not real names throughout the discussion to maintain confidentiality.
- During the focus group discussion, we ask that you not use real names or anything that would identify others.
- We ask each participant to keep what it said in this group to themselves and not gossip about other people's contributions.
- The information gathered will not affect or interfere with your access to health services at any facility or in your community.
- The focus group discussion will be recorded to ensure that it is carried out as planned, and to help us in supplementing our written notes and ensuring their accuracy.
- The focus group discussion will last approximately 1.5 hours.

PRIMARY QUESTIONS	FOLLOW-UP QUESTIONS (PROBES)
**1. EXPERIENCE AND PERSPECTIVES OF COVID-19**<br>*First, we would like to talk about COVID-19.*	
How would you define/describe COVID-19?	***PROBE for***<br>• Contagious illness<br>• Flu-like and people can feel sick<br>• Some people do not feel sick<br>• Can be deadly<br>• There is a vaccine to help prevent COVID-19<br>***Read formal definition of COVID-19***<br>*(COVID-19, an illness caused by the coronavirus, is like the flu and can cause a person's body to feel sick. Most people who have the virus may have a cough, fever, chills (feeling cold) or body aches.)*
Where do you get your information about COVID-19 from? Did you feel like you understand what COVID-19 is and have enough information about how it spreads, ways to protect yourself from getting it, how to access vaccinations, etc.?	***PROBE for***<br>• TV, radio, friends, family, internet, social media<br>• Do you trust the information that you are getting about COVID-19? What is a trustworthy source of this information?
**2. GENERAL IMPACT OF COVID-19**<br>*Next, we would like to talk to you about the impact of COVID-19 and the associated restrictions on your lives, specifically. By COVID-19 associated restrictions, we are referring to national lockdowns, travel restrictions, and quarantine/isolation periods. Let’s talk about the effects that you might have experienced at home and work as well as with family/friends and then we are going to talk specifically about the school closures.*	
How did your work life change due to COVID-19-and COVID-19 related restrictions?	***PROBE for***<br>• Changes in job (loss of job, new job)<br>• Changes in finances<br>• Changes in work method (remote/virtual, hybrid)
How did your home life change due to COVID-19-and COVID-19 related restrictions?	***PROBE for***<br>• Interaction with children<br>• Interaction with partner<br>• Interaction with others in the home<br>• Changes in people living in household<br>• Moving/relocating<br>• Illness/death<br>• Access to services- health? Other support services?

PRIMARY QUESTIONS	FOLLOW-UP QUESTIONS (PROBES)
	● Finances (ability to afford/pay for household goods/food)<br>● Access to food<br>● Access to parks or recreation facilities
Did you or anyone in your house get sick/fall ill with COVID-19?	● Who got sick?<br>● Did they need to go to the hospital? What was that experience like?<br>● If at home, did they isolate away from others?<br>● What was the experience in the house like?<br>● How did you manage or cope with the situation?<br>● How are those that got COVID-19 feeling now?
When you think about the COVID-19 pandemic and the associated restrictions, what were the main challenges you experienced in your life?	***PROBE for***<br>● Economic/financial<br>● Illness or death (self, family)<br>● Social relationships<br>● Romantic relationships<br>● Lifestyle and wellbeing priorities<br>● Children not being at school<br>● Challenges with family<br>● Managing daily living activities<br>● Managing health and wellbeing
When you think about the COVID-19 pandemic and the associated restrictions, what aspects of your life became easier or more enjoyable?	***PROBE for***<br>● Increased sense of community spirit experienced<br>● Break from routine<br>● More free time<br>● More time with family<br>● Nothing
**3. IMPACT OF COVID-19 AND RESTRICTIONS ON CHILDREN'S SCHOOLING AND EDUCATION**<br>*Now we will focus on the impact of COVID-19 and related restrictions on your child's experience in school.*	
3.1 What was school like for your child prior to COVID-19?	*Allow participants to talk about their individual experiences and see what common themes emerge.*<br>***PROBE for***<br>● Amount of time in school<br>● Travel to school<br>● Schooling hours

PRIMARY QUESTIONS	FOLLOW-UP QUESTIONS (PROBES)
	● Number of students (total school population and class sizes)<br>● Interaction with other students<br>● Interaction with teachers<br>● Materials needed<br>● Types of assignments<br>● Quality of education / teaching<br>● Benefits of school?<br>● Challenges related to school?
Please describe when and how your child's school closed at the beginning of the COVID-19 related restrictions.	***PROBE for***<br>● When (month/year) did your child's school close?<br>● How were you notified? By whom?<br>● How quick was the transition?<br>● Who supported your child during the transition?
How did your child's experience of "going to school" change after in-person school closed?	***PROBE for***<br>● Amount of time 'in school'<br>● Method of education (remote/virtual, no schooling, home schooled, etc.)<br>● Schooling hours<br>● Number of students (total school population and class sizes)<br>● Interaction with other students<br>● Interaction with teachers<br>● Materials needed<br>● Types of assignments<br>● Quality of education / teaching
What were some of the challenges **your child experienced** with transitioning to remote/virtual/at home schooling?	***PROBE for***<br>● Access to materials needed for schoolwork<br>● Ability to focus on schoolwork<br>● Interest in school<br>● Different ways of teaching and learning<br>● Support received at home for your child to complete their schoolwork/learning activities
What were some of the challenges **you experienced** due to your child transitioning to remote/virtual/at home schooling?	***PROBE for***<br>● Change in routine<br>● Time spent supporting child with school assignments and technology<br>● Access to materials needed<br>● Ability to focus<br>● Quality of education / teaching

PRIMARY QUESTIONS	FOLLOW-UP QUESTIONS (PROBES)
Did your child have all of the materials (paper/pens, gadgets, etc.) and resources (internet connection, TV broadcast, etc.) that they needed to make the transition to remote/virtual/at home schooling?	***PROBE for***<br>● Please explain<br>● How did they get the materials?<br>● Did you spend your own money on any materials or resources?<br>● Did the school or another organization provide any materials?<br>● Did your child have to share the materials or resources with any other household members?
During the time your child's school was closed, did anyone from their school ever come to your home to check in on your child and how their schoolwork was going?	***PROBE for***<br>● If yes, who came to your house?<br>o What did they do during their visit?<br>o Did they show you and your child how to use any new materials or gadgets needed for school?<br>o Was the visit helpful?<br>● If no, was someone from school supposed to visit your child at home?
How did you support your child at home with their remote/virtual/at home schooling?	***PROBE for***<br>● Assisting child with school (homework/modules)<br>● Assisting child with technology for remote/virtual schooling<br>● Helping them to stay focused<br>● Explaining academic work<br>How comfortable were you providing this support?<br>Did other people (siblings, other family members, friends) also provide support to your child?<br>Did your child receive enough support at home to complete their schoolwork?
How did your child's academic performance change after in-person schooling closed?	***PROBE for***<br>● Why do you think your child's academic performance increased, decreased or stayed the same?<br>● Do you feel that your child learned more or less in the remote/virtual/at home school environment compared to in-person?
Aside from your child's academic classes, did they take part in other types of enrichment and/or support programs at school prior to COVID-19? How did the	***PROBE for***<br>● Pre/post school programs<br>● Nutrition/feeding programs<br>● Guidance counselling

PRIMARY QUESTIONS	FOLLOW-UP QUESTIONS (PROBES)
programs and/or your child's participation change at the beginning of the COVID-19 school closures?	● Academic tutoring (subject-specific or general)<br>● Coaching for academic competitions<br>● School trips/field trips<br>● Sports<br>● Arts<br>● Others
Did you take part in any school activities as your child's caregiver prior to COVID-19? How did your participation / involvement change at the beginning of the COVID-19 school closures?	***PROBE for***<br>● Parent-Teacher Association (PTA)<br>● Attending or volunteering at school programs (plays, fairs, competitions, etc.)<br>● Supporting the feeding program<br>● Other activities
Please describe how your child's new remote/virtual/home schooling experience changed over time.	***PROBE for***<br>● Changes in teaching methods used<br>● Changes in technology utilized<br>● Changes in hours<br>● Changes in types of assignments<br>● Changes in organization and structure<br>● Changes in quality of education<br>● Changes in the amount of support your child needed<br>● Overtime, did it improve overall or not?
Thank you for your contributions so far. We have asked about the impact of the school closures on your child's life and now we would like to focus on the impact on their emotional and physical health and well-being specifically.	
How did your child's mood and emotions change after their **school closed** due to COVID-19?	***PROBE for***<br>● Changes in any of the following emotions:<br>o Happiness<br>o Sadness<br>o Fear<br>o Anger<br>o Anxiety<br>o Loneliness<br>● How did your child cope with these changes in your mood and emotions?<br>o Activities<br>o Self-care<br>o Support from family<br>o Support from friends

PRIMARY QUESTIONS	FOLLOW-UP QUESTIONS (PROBES)
How did your child's physical health change after their **school closed** due to COVID-19?	***PROBE for***<br>● Changes in physical exercise<br>● Changes in nutrition<br>● Changes in drinking habits<br>● Changes in sleep<br>● Exposure to illnesses/getting sick<br>● Feeling healthy or not
**4. TRANSITION BACK TO SCHOOL (reopening)**<br>*Now we want to hear about your experience with your child's transitioning back to in-person schooling after the school closures ended.*	
Please describe the transition of your child returning back to school.	***PROBE for***<br>● When did your child's school reopen to in-person schooling?<br>● How long was your child away from in-person learning?<br>● When were you told the school was reopening for in-person schooling? How quick was the transition back?<br>● COVID-19 safety measures<br>● Hybrid vs. Fully in-person?
How did you feel about your child going back to school in person?	***PROBE for***<br>● Excited/happy<br>● Anxious<br>● Fearful<br>● Sad<br>***Why?***
How did your child feel about going back to school in person?	***PROBE for***<br>● Excited/happy<br>● Anxious<br>● Fearful<br>● Sad<br>***Why?***
Is there anything that you would have changed about the transition back to in-person schooling?	***PROBE for***<br>● New school schedule and methods of teaching (In-person, hybrid, non-hybrid)<br>● Slower or quicker transition back<br>● Vaccination requirements<br>● COVID-19 precautions in schools (masks, temperature checks, hand sanitizer stations, etc.)<br>● Travel/transport going to school and back

PRIMARY QUESTIONS	FOLLOW-UP QUESTIONS (PROBES)
Since your child has returned to in-person school, have you noticed a difference in the quality of in-person teaching and their educational experience compared to remote/virtual/at home schooling?	***PROBE for***<br>● Quality of teaching<br>● Quality of classes<br>● Quality of assignments<br>● Why do you think this is?
Again, we would like to hear from you about the different emotions and feelings your child experienced related to school closures, in this case the reopening and transition back to in-person schooling.	
How has your child's mood and emotions changed after the transition **back to in-person schooling**?	***PROBE for***<br>● Changes in any of the following emotions:<br>o Happiness<br>o Sadness<br>o Fear<br>o Anger<br>o Anxiety<br>● How are you coping with the changes in your mood and emotions?
How has your child's physical health changed after the transition **back to in-person schooling**?	***PROBE for***<br>● Changes in physical exercise<br>● Changes in nutrition<br>● Changes in drinking habits<br>● Changes in sleep<br>● Exposure to illnesses/getting sick<br>● Feeling healthy or not<br>● COVID-19 safety precautions
**5. IMPROVEMENT IDEAS**<br>*Now we are interested in hearing your suggestions for how the transitions from in-person schooling to remote schooling and then back to in-person school could have been improved.*	
How could the transition from **<u>in-person schooling to remote/virtual/at home schooling</u>** (at the beginning of COVID-19) have been better?	***PROBE for***<br>● For you<br>● For your child<br>● Slower/faster transition<br>● Ensure all students have materials they need for remote/virtual/at home schooling<br>● Support from school<br>● Support from teachers
How could the transition **<u>back</u>** **<u>to in-person schooling</u>** have been better?	***PROBE for***<br>● For you<br>● For your child<br>● Slower/faster transition<br>● Ensure all students have materials they need for in-person school

PRIMARY QUESTIONS	FOLLOW-UP QUESTIONS (PROBES)
	• Support from school<br>• Support from teachers
How do you think the changes in your child's schooling due to COVID-19 will impact your child's future?	***PROBE for***<br>• Academic achievements<br>• Mental health<br>• Physical health<br>• Social health
What emotional, academic, and/or social support would help address the continued impact of COVID-19 pandemic and the associated school closures on your child's life?	***PROBE for***<br>• Emotional support from spouse/partner<br>• Emotional support from friends or other parents<br>• Academic support for child(ren) from school<br>• Child(ren) provided with the materials/technology needed to complete academic work<br>• More flexible work schedule (hours, location)<br>• More opportunities to socialize with friends/family
We are at the end of the discussion. Do either of you have anything to add? Or is there anything you forgot to tell us?	
